# Comparative proteomic analysis of different stages of breast cancer tissues using ultra high performance liquid chromatography tandem mass spectrometer

**DOI:** 10.1371/journal.pone.0227404

**Published:** 2020-01-16

**Authors:** Abdullah Saleh Al-wajeeh, Salizawati Muhamad Salhimi, Majed Ahmed Al-Mansoub, Imran Abdul Khalid, Thomas Michael Harvey, Aishah Latiff, Mohd Nazri Ismail

**Affiliations:** 1 Anti-Doping Lab Qatar, Doha, Qatar; 2 Analytical Biochemistry Research Centre (ABrC), Universiti Sains Malaysia, USM, Penang, Malaysia; 3 School of Pharmaceutical Sciences, Universiti Sains Malaysia, USM, Penang, Malaysia; 4 Seberang Jaya Hospital, Perai, Penang, Malaysia; H Lee Moffitt Cancer Center and Research Institute, UNITED STATES

## Abstract

**Background:**

Breast cancer is the fifth most prevalent cause of death among women worldwide. It is also one of the most common types of cancer among Malaysian women. This study aimed to characterize and differentiate the proteomics profiles of different stages of breast cancer and its matched adjacent normal tissues in Malaysian breast cancer patients. Also, this study aimed to construct a pertinent protein pathway involved in each stage of cancer.

**Methods:**

In total, 80 samples of tumor and matched adjacent normal tissues were collected from breast cancer patients at Seberang Jaya Hospital (SJH) and Kepala Batas Hospital (KBH), both in Penang, Malaysia. The protein expression profiles of breast cancer and normal tissues were mapped by sodium dodecyl sulfate polyacrylamide gel electrophoresis (SDS-PAGE). The Gel-Eluted Liquid Fractionation Entrapment Electrophoresis (GELFREE) Technology System was used for the separation and fractionation of extracted proteins, which also were analyzed to maximize protein detection. The protein fractions were then analyzed by tandem mass spectrometry (LC-MS/MS) analysis using LC/MS LTQ-Orbitrap Fusion and Elite. This study identified the proteins contained within the tissue samples using *de novo* sequencing and database matching via PEAKS software. We performed two different pathway analyses, DAVID and STRING, in the sets of proteins from stage 2 and stage 3 breast cancer samples. The lists of molecules were generated by the REACTOME-FI plugin, part of the CYTOSCAPE tool, and linker nodes were added in order to generate a connected network. Then, pathway enrichment was obtained, and a graphical model was created to depict the participation of the input proteins as well as the linker nodes.

**Results:**

This study identified 12 proteins that were detected in stage 2 tumor tissues, and 17 proteins that were detected in stage 3 tumor tissues, related to their normal counterparts. It also identified some proteins that were present in stage 2 but not stage 3 and vice versa. Based on these results, this study clarified unique proteins pathways involved in carcinogenesis within stage 2 and stage 3 breast cancers.

**Conclusions:**

This study provided some useful insights about the proteins associated with breast cancer carcinogenesis and could establish an important foundation for future cancer-related discoveries using differential proteomics profiling. Beyond protein identification, this study considered the interaction, function, network, signaling pathway, and protein pathway involved in each profile. These results suggest that knowledge of protein expression, especially in stage 2 and stage 3 breast cancer, can provide important clues that may enable the discovery of novel biomarkers in carcinogenesis.

## Introduction

Breast cancer has been reported to be the fifth most common cause of death among women and one of the most widely diagnosed cancers afflicting women globally [1–3]. The impact of breast cancer’s prevalence is illustrated to affect more than 1.3 million women each year, and 1 in 8 women at some point in their lives [4]. Similarly, breast cancer is the second leading cancer in the United States and was approximated to cause about 14% of all cancer-related deaths. In the past few decades, the volume of deaths from breast cancer prompted a rapid effort to improve screenings, leading to an upward trend in breast cancer diagnoses that some predicted to continue in the future [5].

Breast cancer is reported most frequently in specific parts of the world, including developed countries in Northern Europe and North America, Mediterranean and South America, and impoverish countries in Africa and Asia [6]. With references to these expectations, Ziegler et al. [7] postulated that in the coming year, breast cancer diagnoses will affect 230,000 women, of which 40,000 may lose their lives because of this cancer. Historically, there are few reported cases of breast cancer in Asian countries, but now they are witnessing an increase in diagnoses [8–10].

Several reports have questions about the incidence and prevalence of breast cancer in Asian countries that affected almost every 1 in 19 women [11, 12]. In Malaysia, breast cancer is one of the most common types of cancer among women, with an estimated age-standardized rate (ASR) of approximately 38.7 per 100,000, with 5410 new cases each year [13, 14]. However, the number of Malaysian women at risk for breast cancer may be much higher than what is reported currently because some patients still seek traditional therapy and may not document their breast cancer cases in a conventional way [15].

Among breast cancer, ductal carcinoma is the most prevalent and the most life-threatening type of breast cancer [16]. Invasive ductal carcinoma (IDC) starts in the lactiferous duct, penetrates the duct tube, and then attacks the nearest breast tissue. From there, it can metastasize to other parts of the body through the lymph and blood systems (metastasis) [17]. Thus, it is critical that researchers devote attention to the identification of markers that discriminate tumorigenic from normal cells and may differentiate between different stages of malignant pathology. This essential task is made possible by proteomics, which analyzes protein expression profiles and maps the differences between the profiles of breast cancer tissue and the profiles of adjacent normal tissues. Since breast cancer patients have a promising prognosis only if their disease is diagnosed early, before advancing to an extent that modern medicine cannot address, there is a need to find dependable tissue target of breast cancer. The identification of potential proteins among breast cancer patients could aid early detection, treatment, monitoring, identify carcinogenesis, and prognosis of breast cancer stages. Mass spectrometry-based platforms have become an essential component for rapidly testing and qualifying a large number of candidate biomarkers for further development and validation of breast cancer [18–20]. Recently, Devlin et al. [21] were used liquid chromatography coupled to an LTQ-XL linear ion trap mass spectrometer to highlight novel roles of protein septin 9 in the pathogenesis of breast cancer. However, there is a persisting need to discover a reliable biomarker that can detect breast cancer reliably and at an early stage. In this study, proteomic profiling was carried out on samples of both breast cancer and adjacent normal tissues using advanced techniques such as the LTQ-Orbitrap, the GELFREE fraction system, and bioinformatics PEAKS software, with the aim of identifying protein biomarkers that may be employed in the diagnosis and/or treatment of breast cancer.

## Materials and methods

### Study ethics and sample collection

Ethics approval for the collection of breast tissue samples was obtained from the Human Ethical Committee of the Ministry of Health Malaysia and the Human Ethical Clearance Committee of Universiti Sains Malaysia (Reference No. USMKK/PPP/JEPeM "211.3.15"). The study was designed and conducted in accordance with the ethical principles, and all participants signed informed consent forms to allow the researcher to take the breast tissues for the experimental procedures (see S1 Appendix). In the current study, all consent forms were only self-signed by the patients who agreed to be part of this research study for the use of their tissues in the experimental procedures during their Mastectomy and if needed just confirmed from their legal representative (relatives).

In this study, all samples were collected through a surgical procedure and were not biopsies nor postmortem. Through a surgical procedure, a total of eighty samples comprised forty pairs of breast tumor and adjacent normal tissues from the same patient were collected from January 2010 till February 2012 in the present study. Breast samples were collected at Seberang Jaya Hospital (SJH) and Kepala Batas Hospital (KBH) in Penang, Malaysia. The tissues were obtained from middle-aged and elderly women (32–78 years). The collected samples were evaluated and grouped in the analysis according to histopathology report after diagnosis. All tumor tissues were collected by removal the whole affected breast tissues through surgical procedure (Mastectomy). The breast carcinoma typing and grading were confirmed by a pathologist according to the World Health Organization criteria [22, 23]. In the control group, adjacent tissues were taken from the non-afflicted tissue of breast cancer patients, sampled from a distance of at least 10 cm from the tumor. Then, the tissue samples were preserved at -80°C. Before the protein extraction step, the samples were left to thaw at room temperature and rinsed with cold distilled water. A scalpel was used to remove the fatty coating from each sample. The remaining tissue was chopped into very small pieces, weighed, and placed in labelled microcentrifuge tubes for the analysis stage. All clinical information was obtained from archives of case history from SJH and KBH that included demographic details, medical history, notes from clinical examinations, histopathology type of breast cancer (such as, the stage of breast cancer at presentation), the technique of diagnosis, treatment offered at diagnosis, therapy and diagnostic investigation, respectively. The clinical-pathological characteristics of breast cancer patients are listed in Table 1. This research represents a critical step toward fulfilling the exigent need to differentiate and detect more sensitive markers for the early detection and diagnosis of breast cancer. Thus, the study carried on the three major ethnic groups namely Malays, Chinese and Indians that exist in Malaysia. This research was in the same line for breast cancer prevention and control programs in Malaysia which could hopefully help oncology professionals in the planning of practical preventive research strategies [24–26]. The ethnicity of the patients was based on the documentation from the hospital which was self-identified by the patients.

**Table 1:** Clinical pathological characteristics and molecular sub-type of stage 2 and stage 3 of breast cancer patients.

Patient No.	Patient ID.	Stage	Histopathological Diagnosis	ER/PR/HER2	TNBC	ANT
**1**	**P2**	2	IDC	+/+/-	--	+
**2**	**P3**	2	IDC	+/-/+	--	+
**3**	**P4**	2	IDC	+/+/-	--	+
**4**	**P5**	2	IDC	+/+/-	--	+
**5**	**P7**	2	IDC	+/+/+	--	+
**6**	**P10**	2	IDC	+/+/-	--	+
**7**	**P20**	2	IDC	-/+/+	--	+
**8**	**P21**	2	IDC	+/+/-	--	+
**9**	**P31**	2	IDC	+/+/-	--	+
**10**	**P32**	2	IDC	+/+/+	--	+
**11**	**P40**	2	IDC	+/+/-	--	+
**12**	**P41**	2	IDC	+/+/+	--	+
**13**	**P42**	2	IDC	+/+/-	--	+
**14**	**P64**	2	IDC	+/+/+	--	+
**15**	**P65**	2	IDC	+/+/-	--	+
**16**	**P74**	2	IDC	+/+/-	--	+
**17**	**P78**	2	IDC	+/-/-	--	+
**18**	**P82**	2	IDC	+/+/+	--	+
**19**	**P99**	2	IDC	+/+/-	--	+
**20**	**P113**	2	IDC	+/-/+	--	+
**21**	**P114**	3	IDC	+/-/+	--	+
**22**	**P115**	3	IDC	+/-/-	--	+
**23**	**P137**	3	IDC	+/-/-	--	+
**24**	**P138**	3	IDC	+/++	--	+
**25**	**P139**	3	IDC	+/-/+	--	+
**26**	**P147**	3	IDC	+/-/+	--	+
**27**	**P148**	3	IDC	-/-/+	--	+
**28**	**P150**	3	IDC	-/-/+	--	+
**29**	**P156**	3	IDC	+/-/+	--	+
**30**	**P157**	3	IDC	+/-/-	--	+
**31**	**P160**	3	IDC	+/-/-	--	+
**32**	**P161**	3	IDC	+/-/+	--	+
**33**	**P165**	3	IDC	+/-/-	--	+
**34**	**P168**	3	IDC	+/+/+	--	+
**35**	**P176**	3	IDC	+/+/+	--	+
**36**	**P178**	3	IDC	+/+/+	--	+
**37**	**P180**	3	IDC	+/+/+	--	+
**38**	**P191**	3	IDC	+/+/+	--	+
**39**	**P198**	3	IDC	+/-/+	--	+
**40**	**P201**	3	IDC	+/-/-	--	+

IDC, Invasive ductal carcinoma; ER/PR/HER2, estrogen receptor/ progesterone receptor/ human epidermal growth factor receptor 2; TNBC, Triple-negative breast cancer; ANT, adjacent non-tumor breast tissue.

### Protein extraction and processing

Liquid nitrogen was used to grind all tissues individually and then the tissues were disrupted with a glass homogenizer [27]. All tissue samples were run individually in triplicate. The proteins were extracted with lysis buffer containing 25 mM Tris, 150 M NaCl, 5 mM EDTA, and 1% CHAPS, with the pH adjusted to 7.4. The homogenized tissue was then subjected to vortexing (about 30 seconds) and centrifugation at 1500 x g (Thermo Fisher Scientific, Osterode, Germany) at 4°C for 10 minutes. The eluted proteins were dialyzed with 7000-MW cut-off (MWCO) SnakeSkin pleated dialysis tubing (Thermo Scientific Co., Rockford, IL, USA) and sealed firmly. Cool dialysis buffer (50 mM ammonium bicarbonate, pH 7.5) was used to homogenize the tissues at 4°C. The dialysis buffer was changed every 48 hours. The homogenates were placed in microcentrifuge tubes and kept at -80°C until analysis. The protein concentrations of all samples were determined by the Bradford assay method [28]. Briefly, an amount of 5 μL of each sample was mixed with 250 μL of Bradford reagent in a 96-well plate and then incubated at room temperature for 15 minutes to measure the total protein concentration in each sample following the manufacturer’s instructions obtained from Bio-Rad Laboratories (Hercules, CA, USA). A serial dilution ranging from 0.0 to 2 mg/mL of bovine serum albumin (BSA) was used to generate a calibration curve. Thereafter, the absorbance of both the samples and standard was measured at 595 nm. The average concentration of each sample was calculated from the calibration curve.

### Protein fractionation by GELFREE 8100 system

The GELFREE 8100 fractionation system (Expedeon, CA, USA) was chosen for protein fractionation using 10% Tris-acetate cartridge [29]. The complexity of the protein extracts was reduced with molecular weight fractionation, applied to 150 μg portions of the protein extracts. Furthermore, the electrophoretic separation was performed using SDS-PAGE material in a tubular environment. Finally, GELFREE was used to fractionate the proteins in the liquid phase. This process yielded 12 liquid fractions (separated by MWCO), these fractions were aggregated into four groups, each comprising three fractions, and the total protein concentration was measured for each combined fraction. A 50 μg protein sample from each combined fraction was frozen for 30 minutes at -80°C. Thereafter, it was freeze-dried overnight in preparation for the analysis. S1 Table displays fractionation conditions of the GELFREE 10% Mass Cartridge Kit (see S1 Appendix).

### Image analysis

In this study, SDS-PAGE was employed with 12.5% resolving slab gels to evaluate the success of the GELFREE fractionation and quality of separation. The first and last lanes of each gel were loaded with the Precision Plus Protein^™^ SDS-PAGE Standards (Bio-Rad Laboratories Inc., Hercules, CA, USA). The gel scan was performed with GeneSys G: Box Chemi-XX8 image analyzer (Syngene, Funakoshi Co. Ltd., Japan). The GeneSys program was used to capture the image (GeneSys V1.3.9.0 -Syngene). SDS-PAGE was used to evaluate the resolution of the GELFREE fractions, to verify the quality of the fractionation process, and to demonstrate successful separation. If the proteins had been separated adequately, different bands would appear in different fractions.

### Protein digestion by in-solution tryptic digestion

To prepare the samples for nanoflow UHPLC separation and analysis using high-resolution Mass Spectrometry (MS) and Tandem Mass Spectrometry (MS/MS), 50 μg of protein from each GELFREE-combined fraction was retained subjected to the In-Solution Tryptic Digestion method as reported by Ru et al. [30]. Each of the combined fraction was re-suspended with 6M guanidine-HCL/25 mM ammonium bicarbonate (NH_4_HCO_3_) at pH 8.5, and then reduced with 250 μL of 1 mg/mL dithiothreitol (DTT, 200 mM) in 25 mM NH_4_HCO_3_. The sample was incubated at 55°C for 30 minutes, alkylated with 500 μL of 1 mg/mL iodoacetamide (IAA, 200 mM) in 25 mM NH_4_HCO_3_, and incubated in a dark room at 55°C for 15 minutes. The reduced and alkylated protein samples were concentrated and desalted using buffer exchange with 25 mM NH_4_HCO_3_ in a spin-column with a molecular cut-off of 3 kDa, run 3 times at 3500 x g (Eppendorf AG, Hamburg, Germany) for 45 minutes, follow by digestion of the concentrated protein with the addition of 1 μL of reconstituted 1 μg/μL trypsin (ratio 1:50). The reaction was run at 37°C for 18 hours (overnight), after which 0.1 μL of formic acid was added to stop the trypsin reaction.

### Quantitative LC-MS/MS analysis

Prior to LC-MS/MS analysis, the peptide samples were mixed with 100 μL of 0.1% formic acid in deionized water and filtered with a 0.45 μm syringe filter. A 10 μL sample of each peptide was desalted using Zip-Tip C18 tips (Millipore Co., Billerica, MA, USA) according to the manufacturer’s protocol (www.millipore.com). All analyses of LC-MS and MS/MS were performed using a nano UHLPC system coupled with LTQ-Orbitrap Fusion MS (Thermo Scientific Co., San Jose, CA, USA). The chromatographic separation of tryptic-digested peptides was carried out using the easy-column C18 (10 cm, 0.75 mm i.d., 3 μm particles size; Thermo Scientific Co., USA), which was used as the analytical column. Easy-column C18 (2 cm, 0.1 mm i.d., 5 μm particles size; Thermo Scientific Co., USA) was used as the pre-column. A 1 μL of the sample was injected and chromatographically separated at a flow rate of 0.3 μL/min. Two mobile phase running buffers: (A) 0.1% formic acid in deionized water, and (B) acetonitrile with 0.1% formic acid were used and the samples were eluted using a gradient of buffer B, ranging from 5% to 100%, over the course of about 85 minutes. Data was acquired with Xcalibur software version 2.1 (Thermo Scientific Co., San Jose, CA, USA) with a mass tolerance threshold of 5 ppm.

The eluent was sprayed into the mass spectrometer at 2.1 kV (source voltage) at a capillary temperature of 220°C. Peptides were detected using full-scan mass analysis, from m/z 300 to 2,000 at a resolving power of 60,000 (at m/z 400, FWHM; 1-s acquisition), with data-dependent MS/MS analyses (ITMS) triggered by the eight most abundant ions from the parent mass list of predicted peptides. Peptides with single or unassigned charge states were rejected. Collision-induced dissociation (CID) with a collision energy of 35 was used as the fragmentation technique. To perform *de novo* sequencing and database matching, PEAKS software version 7.5 (Bioinformatics Solutions Inc., Waterloo, ON, Canada) was implemented. The MS/MS analysis was carried out using similar resolving power (60,000). The CID was applied with an isolation width of 2 Da, a normalized collision energy of 35, an activation *q* of 0.25, an activation time of 50 ms, and a charge state of 2. Higher-energy Collisional Dissociation (HCD) was applied with an isolation width of 2 Da, normalized collision energy of 35, an activation time of 0.1 ms, and an FT first mass value (m/z) of 100 [31].

### Protein identification

From the raw data generated by LC-MS/MS-LTQ-Orbitrap, protein identities were investigated using the standard PEAKS workflow by using the following parameters: Homo sapiens UniProt reference proteome for the database (http://www.uniprot.org/) released March 2015, trypsin for enzyme, and cysteine carbamidomethylation and methionine oxidation as fixed post-translation modifications (PTM). The PTM variables comprised the phosphorylation sites S, T, and Y. The error tolerance, parent ion and fragment ion were set at 15 ppm and 0.8 Da, respectively. Using LTQ-Orbitrap instrument with a maximum of 2 missed cleavages per peptide and a maximum of 5 variable PTMs per peptide. The protein –10logP was set to ≥20, peptide –10logP was set to ≥15, Peptide Spectrum Match’s false discovery rate (FDR) was set to 1.00%, and the number of unique peptides was set to ≥1.

### Data mining and bioinformatics analysis

#### Gene ontology and pathway enrichment

DAVID webserver was used to analyze the significance of the presence of the detected proteins in different pathways [32]. Specifically, annotations were assessed in the following categories: Gene Ontology terms, GAD and OMIM for diseases, chromosome and cytoband for general annotations, BioCarta, KEGG and Reactome for molecular pathways, SMART and INTERPRO for protein domains, INTACT and BioGrid for protein-protein interactions, the UCSC repository for Transcription Factor enrichment, and CGAP and UP_TISSUE for gene expression enrichment. Then, the significant terms *P<*0.05 and *P≤*0.01 were obtained for each list of proteins in the cancer samples and the adjacent normal samples.

#### Network analysis

The potential interactions across previously-detected proteins were studied with the STRING webserver [33]. The P-value was calculated from the ratio of the number of interactions observed in the set of proteins and the number of interactions predicted in the set while all the sets were more connected than expected. Finally, relevant biological action in these networks was estimated by plotting only the interactions for each gene set (e.g., activation, inhibition, binding, reaction).

#### Cytoscape-based representative models

The lists of molecules were the input of the REACTOME-FI plugin [34], part of the CYTOSCAPE tool [35]. Linker nodes were added to generate a connected network. Then, pathway enrichment was obtained (FDR<0.25) and a graphical model of the participation of the input proteins as well as the linker nodes was generated.

### Statistical analysis

Statistical analysis was performed according to Li et al. [36] and Torres-Luquis et al. [37]. Data were stored in Excel and descriptive statistics were calculated for all the variables. The statistical analyses were performed with SPSS 20.0 software. The presence of the detected proteins in the tumor tissues and not exist in their matched adjacent normal tissues was performed against a chi-square test. Two-sample paired t-test was used to compare tumor and normal breast tissues. Zero was used to signify the lack of intensity if no protein was seen. The most significant proteins were reported. All the values of the percentages found in the tumor samples were found to be statistically *P<*0.05.

## Results

**Fig 1:**
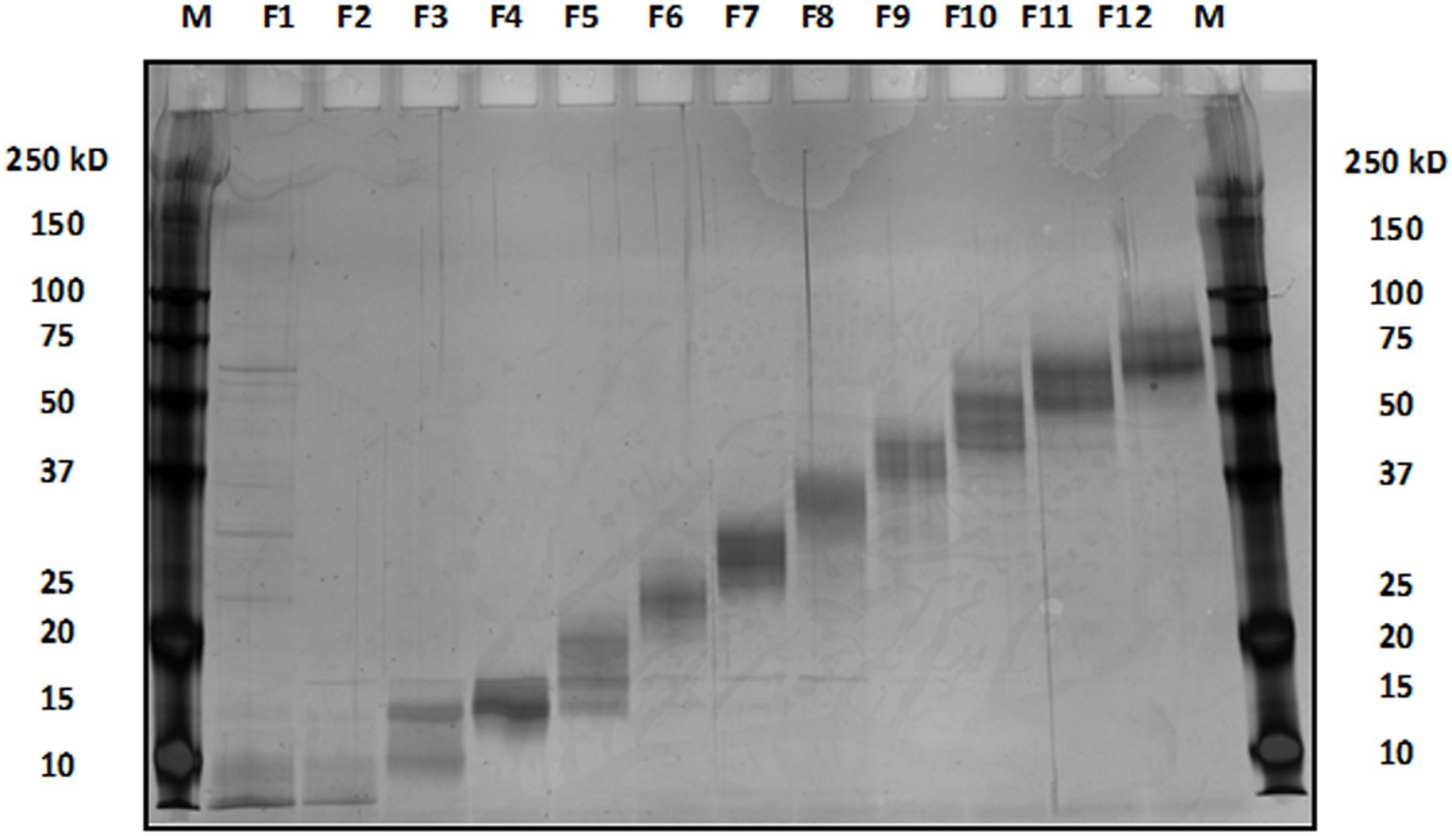
GELFREE protein fraction bands of tissue sample separated by 12% SDS-PAGE.

The protein extracts from tissue samples of both tumor and adjacent normal origins were subjected to molecular weight fractionation via electrophoretic separation through SDS-PAGE material in the tubular environment, all in the liquid-phase (GELFREE). This yielded 12 liquid fractions (separated by MWCO) for each protein extract (Fig 1). In addition, all uncropped and full images of SDS-PAGE for the GELFREE images were added (see S2 Appendix).

Initially, each of these 12 fractions was reduced, alkylated, and subjected to trypsin digestion before nanoflow UHPLC separation and high-resolution MS and MS/MS analysis. Each specimen required a minimum of 12 injections for LC-MS analysis. We decided to investigate the possibility of combining some of these GELFREE fractions to reduce the number of LC-MS analyses required for each tissue specimen. To investigate this possibility, several crude protein extracts were separated by GELFREE and then two experimental protocols were investigated in parallel. In the first protocol, each of the 12 fractions was digested and subjected individually to MS and MS/MS analysis with a nanoflow UHPLC separation. The quality of each of these 12 data sets was evaluated with protein and peptide identifications. In the second protocol, the 12 GELFREE fractions were combined into 4 groups (fractions 1–3, 4–6, 7–9, and 10–12). These combined fractions were digested and evaluated with MS as in the first protocol. Finally, we compared the final protein and peptide profiles that were generated by the two different protocols [38].

After several comparisons, the study combined fractions yielded equivalent results in terms of identification, coverage, and data fidelity as the sum of the individual constituent fractions and we reanalyzed separately. Since the combination strategy did not degrade the quality or usefulness of the protein and peptide identifications combining the 12 initial fractions into 4 groups of 3 fractions, each therefore was adopted as the standard operating procedure, thus reducing the number of analyses per fraction from 12 to 4 (67% reduction in time and instrument resources).

**Fig 2:**
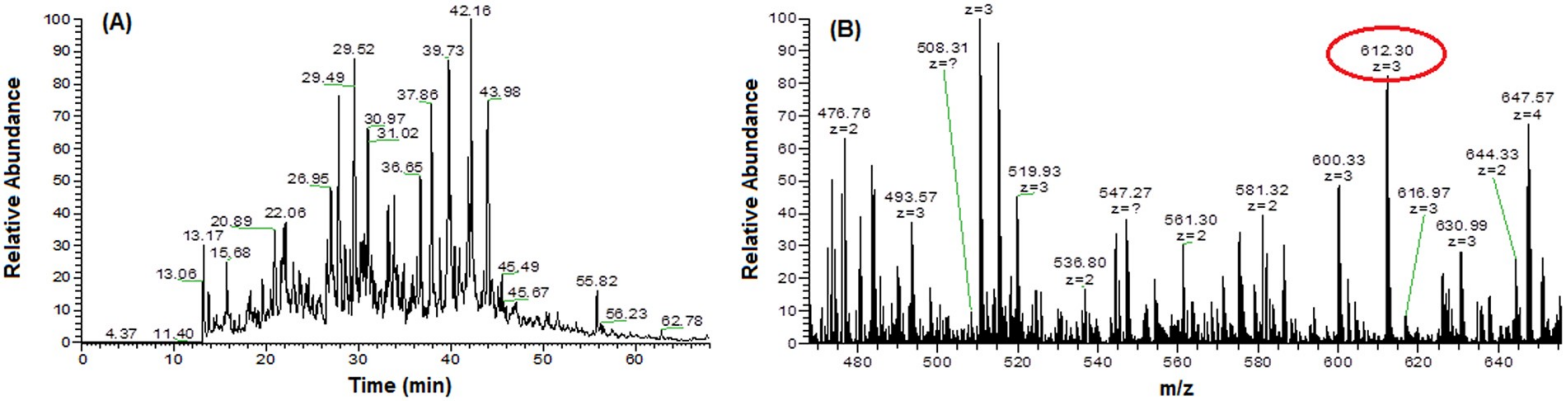
(A) Base peak chromatogram of tissue sample on Orbitrap LTQ-LC/MS–CID activation. (B) Expanded region of a single FTMS full scan, with a mass range of m/z 400–1800, and ion peaks with double or higher charge (612.30, z = 3).

In this study, a comprehensive analysis was performed using the LTQ-Orbitrap Elite with CID fragmentation technique, and PEAKS Client software version 7.5 with the Uniprot (Homo sapiens) database. Fig 2A and 2B show representative MS/MS fragments for the base peak chromatogram of the tissue sample on Orbitrap LTQ-LC/MS–CID activation, and for the expanded region of a single FTMS full scan, with a mass range of m/z 400–1800.

### Protein profiling

Table 2 shows the presence of 12 specific proteins that were identified and detected in stage 2 breast cancer tissues but not in normal tissues. Chi-squared values revealed significant results for 12 of these proteins (*P<*0.05). The list of proteins identified by LC-MS/MS, in stage 2 of breast cancer tissue and their details as shown in PEAKS Software (S2 Table and S3 Appendix). PEAKS provides a number of statistical charts for the peptide and proteins score distributions and Venn diagrams. The figures show the peptides and proteins score distributions, and the Venn diagrams show clearly the non- redundant unique and overlapping of the peptide and proteins numbers in both tumor and the adjacent normal breast tissues for the stages 2 and 3 (see S4 Appendix).

**Table 2:** List of proteins detected in stage 2 breast cancer samples.

No.	Protein Name	Normal		Tumor		Chi^2	P-value	Difference
		N	%	N	%			
**1**	Prolyl 3-hydroxylase 1	0	0%	14	70%	30.77	0.0130	70%
**2**	Peptidyl-prolyl cis-trans isomerase FKBP10	0	0%	12	60%	28.57	0.0356	60%
**3**	CAP-Gly domain-containing linker protein 1	0	0%	12	60%	28.57	0.0356	60%
**4**	Peptidyl-prolyl cis-trans isomerase FKBP9	0	0%	12	60%	28.57	0.0356	60%
**5**	Zinc finger CCCH domain-containing protein 18	0	0%	12	60%	28.57	0.0356	60%
**6**	Immunoglobulin superfamily containing leucine-rich repeat protein	0	0%	12	60%	28.57	0.0356	60%
**7**	MOB kinase activator 1A	0	0%	12	60%	28.57	0.0356	60%
**8**	Protein enabled homolog	0	0%	12	60%	28.57	0.0356	60%
**9**	Collagen alpha-1(V) chain	0	0%	12	60%	28.57	0.0356	60%
**10**	Protein canopy homolog 4	0	0%	12	60%	28.57	0.0356	60%
**11**	Perilipin-4 (PLIN4)	0	0%	12	60%	28.57	0.0356	60%
**12**	Transmembrane emp24 domain-containing protein 10	0	0%	12	60%	28.57	0.0356	60%

N: number of specific identified proteins.

Unit: % percentage of total detection.

Additionally, we found 17 proteins that were detected in stage 3 breast cancer samples compared to the normal samples (Chi-square values indicated significant results for all 17 proteins, *P≤*0.01) as revealed in Table 3 (S4 Table and S5 Appendix).

**Table 3:** List of proteins detected in stage 3 breast cancer samples.

No.	Protein Name	Normal		Tumor		Chi^2	P-value	Difference
		N	%	N	%			
**1**	Golgi resident protein GCP60	0	0%	16	80%	33.33	0.0040	80%
**2**	Eukaryotic peptide chain release factor subunit 1	0	0%	14	70%	30.77	0.0130	70%
**3**	Nucleoside diphosphate kinase 3	0	0%	14	70%	30.77	0.0130	70%
**4**	Deoxynucleoside triphosphate triphosphohydrolase SAMHD1	0	0%	14	70%	30.77	0.0130	70%
**5**	Protein SEC13 homolog	0	0%	14	70%	30.77	0.0130	70%
**6**	Protein enabled homolog	0	0%	14	70%	30.77	0.0130	70%
**7**	Rho GTPase-activating protein 1	0	0%	14	70%	30.77	0.0130	70%
**8**	LEM domain-containing protein 2	0	0%	14	70%	30.77	0.0130	70%
**9**	TAR DNA-binding protein 43	0	0%	14	70%	30.77	0.0130	70%
**10**	V-type proton ATPase subunit E 1	0	0%	14	70%	30.77	0.0130	70%
**11**	Prefoldin subunit 1	0	0%	14	70%	30.77	0.0130	70%
**12**	Coiled-coil domain-containing protein 58	0	0%	14	70%	30.77	0.0130	70%
**13**	Inhibitor of nuclear factor kappa-B kinase-interacting protein	0	0%	14	70%	30.77	0.0130	70%
**14**	DNA-dependent protein kinase catalytic subunit	0	0%	14	70%	30.77	0.0130	70%
**15**	Transmembrane glycoprotein NMB	0	0%	14	70%	30.77	0.0130	70%
**16**	MOB kinase activator 1A	0	0%	14	70%	30.77	0.0130	70%
**17**	MOB kinase activator 1B	0	0%	14	70%	30.77	0.0130	70%

N: number of specific identified proteins.

Unit: % percentage of total detection.

In this study, the protein profiles of stage 2 breast cancer tissues and normal tissues were successfully identified with the proteomics approach. This study yielded high confidence data with high mass accuracy and resolution. In addition, the results produced by the GELFREE combined fractions were equivalent, in terms of protein identifications, coverage, and data fidelity, to the results produced by individual fractions. Based on a series of experiments over several months, it was concluded that such a combination strategy did not degrade the quality or utility of the protein and peptide identifications.

**Fig 3:**
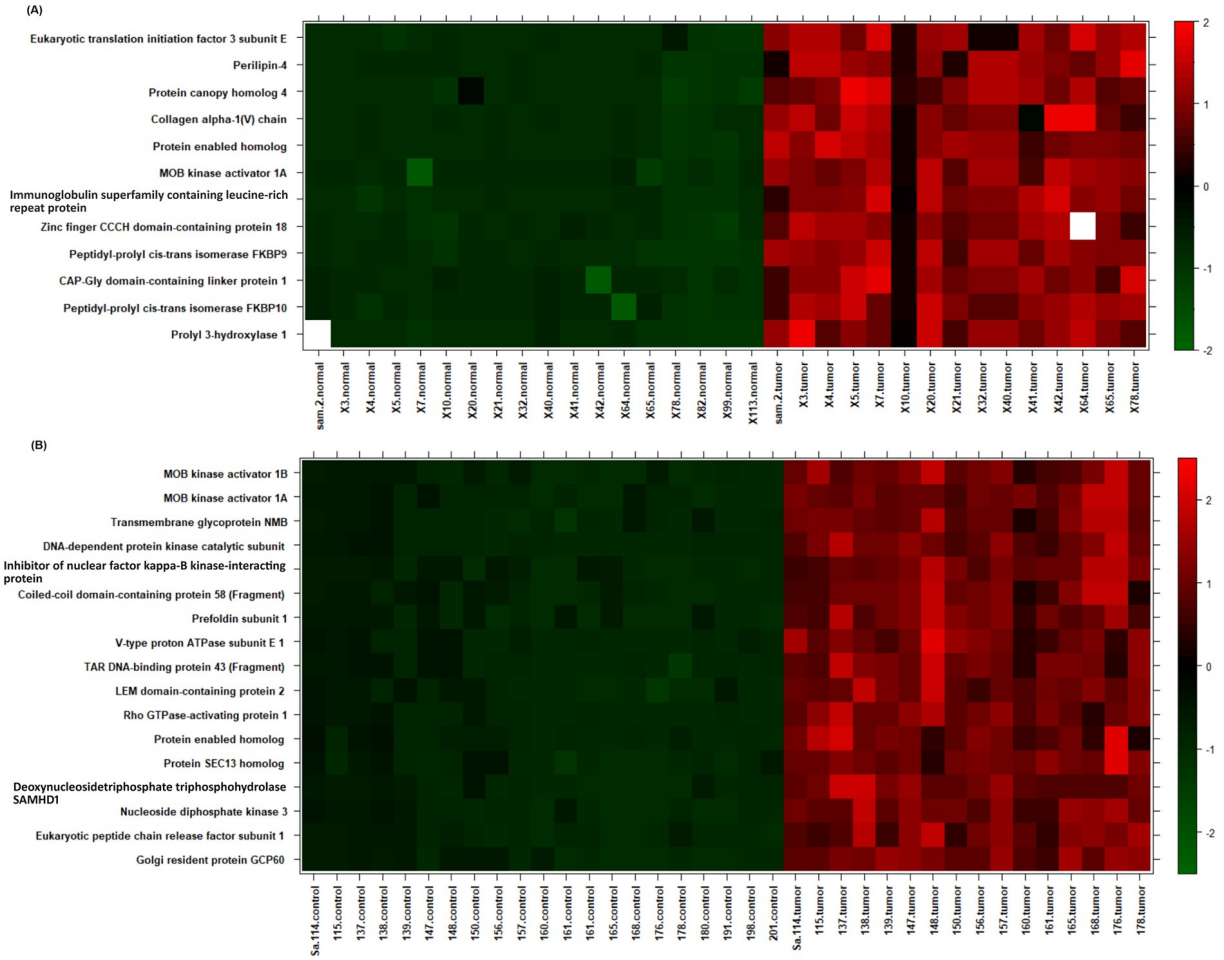
Heat map analysis of the detected proteins in stage 2 and 3 of the tumor and normal breast tissues. Heatmap of significantly different proteins between tumor and normal breast tissues. Samples on x-axis were ordered by tumor or normal group. The color code denotes z-scaled values of proteins signal intensities.

Proteins that showed significant differences between tumor and normal breast tissues were projected on the heatmap using the R statistical package (version 2.14, www.r-project.org/), representing a snapshot summary of the actual intensities of these proteins. Proteins were z-scaled by subtracting their means followed by division by standard deviations (Fig 3).

### Protein pathways in stage 2 breast cancer

**Fig 4:**
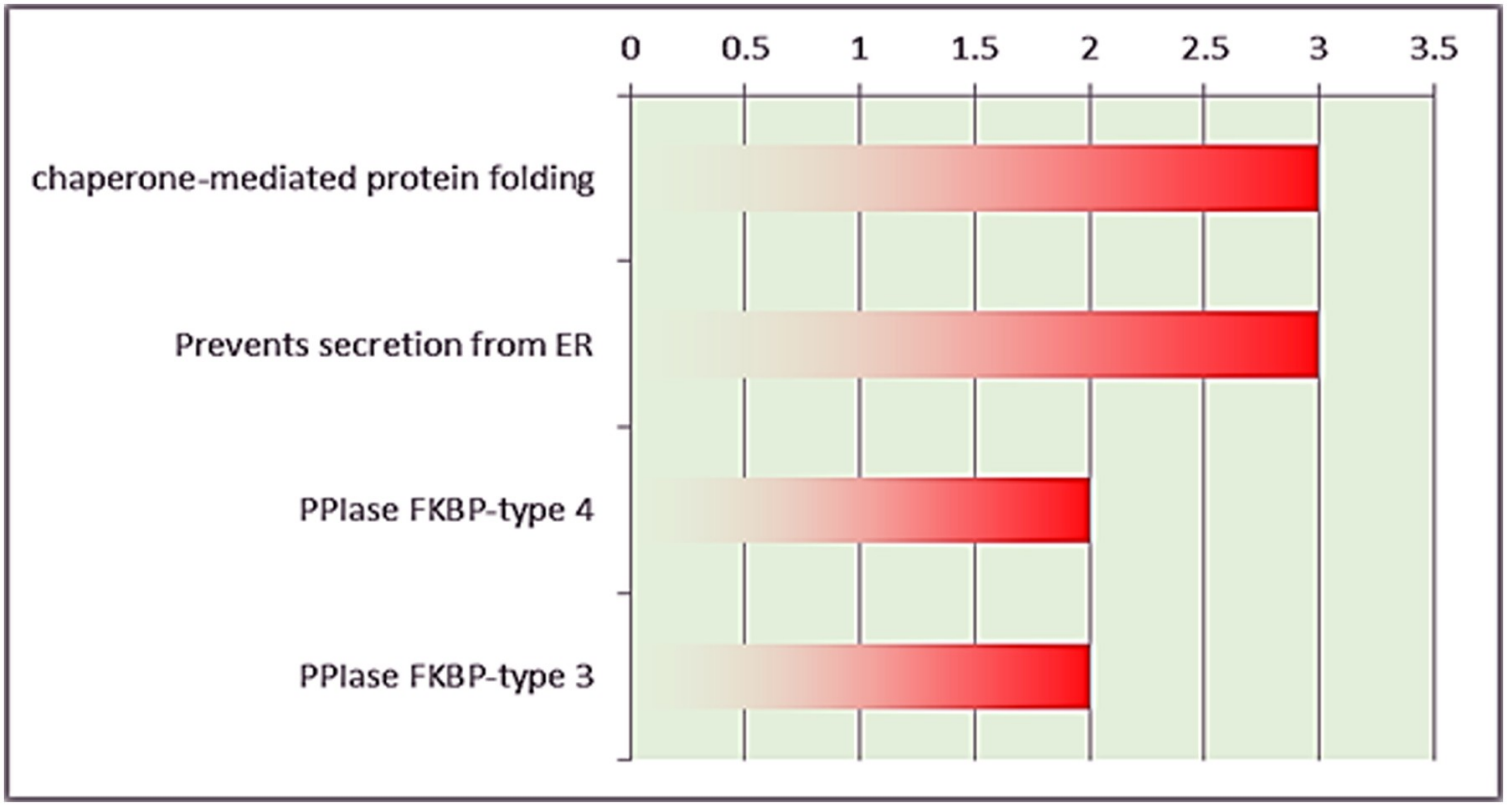
DAVID analysis for stage 2 breast cancer samples.

The DAVID results revealed that several proteins were detected in stage 2 breast cancer act as significant protein folding chaperones (Fig 4). The complete list of proteins identified by LC-MS/MS in stage 2 of breast cancer tissue (S3 Table).

**Fig 5:**
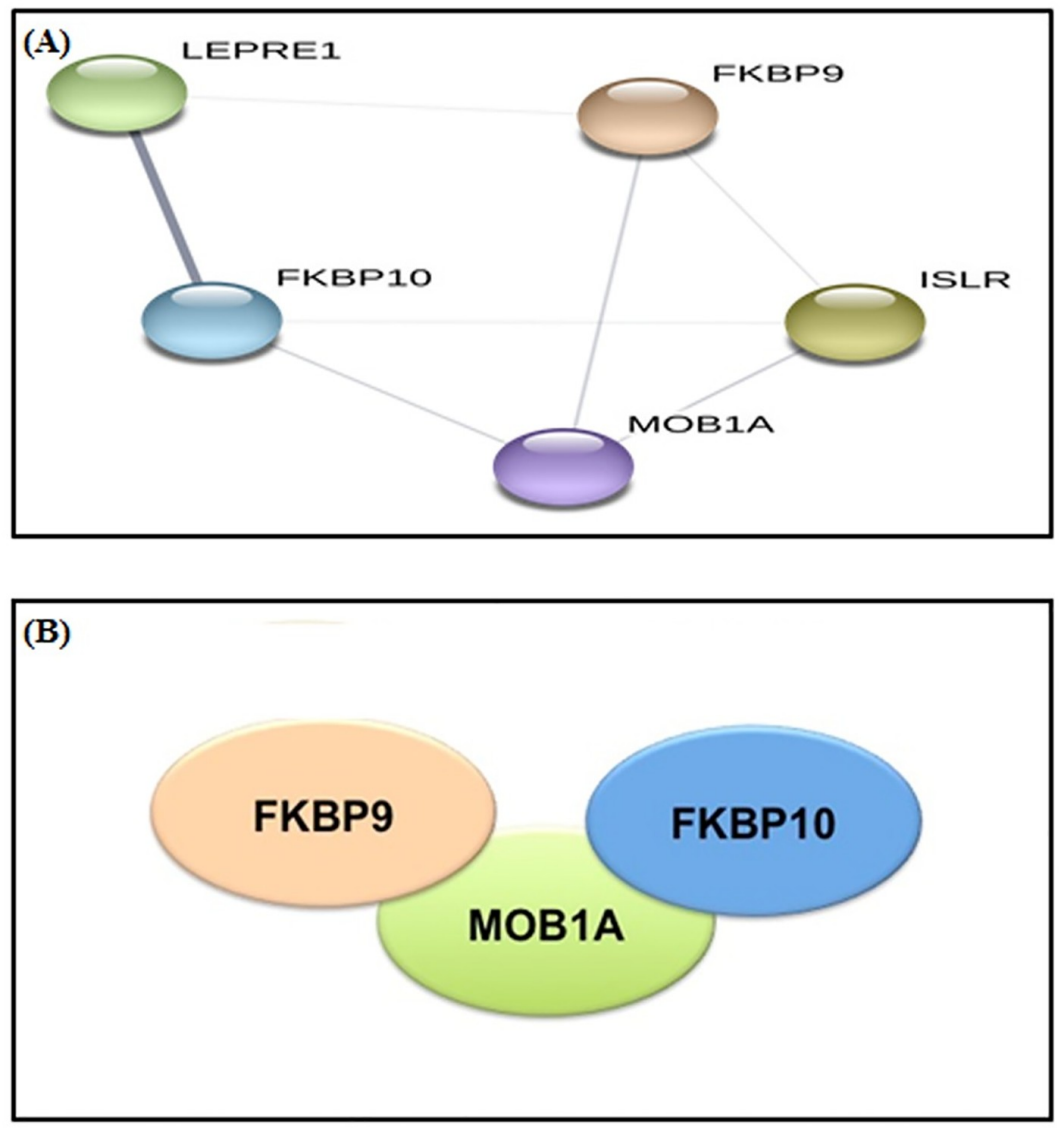
(A) Functional network of proteins in stage 2 breast cancer samples, (confidence level = 0.150). (B) Molecular pathway of proteins as predicted by STRING. Schematic representation of potential protein-protein interactions. This is predicted directly by DAVID, each ellipse represents a protein, and the representation suggests the interaction pathway. In this case, MOB1A interacts independently with FKBP9 and FKBP10.

Fig 5A and 5B show the unique proteins in stage 2 breast cancer constitute a highly interconnected functional network, which suggests a large conserved complex with the involvement of at least three proteins (MOB1A, FKBP9, and FKBP10). It shows a non-directed graph with the functional interactions as predicted by STRING. Each node represents a protein name that is located near each circle.

**Fig 6:**
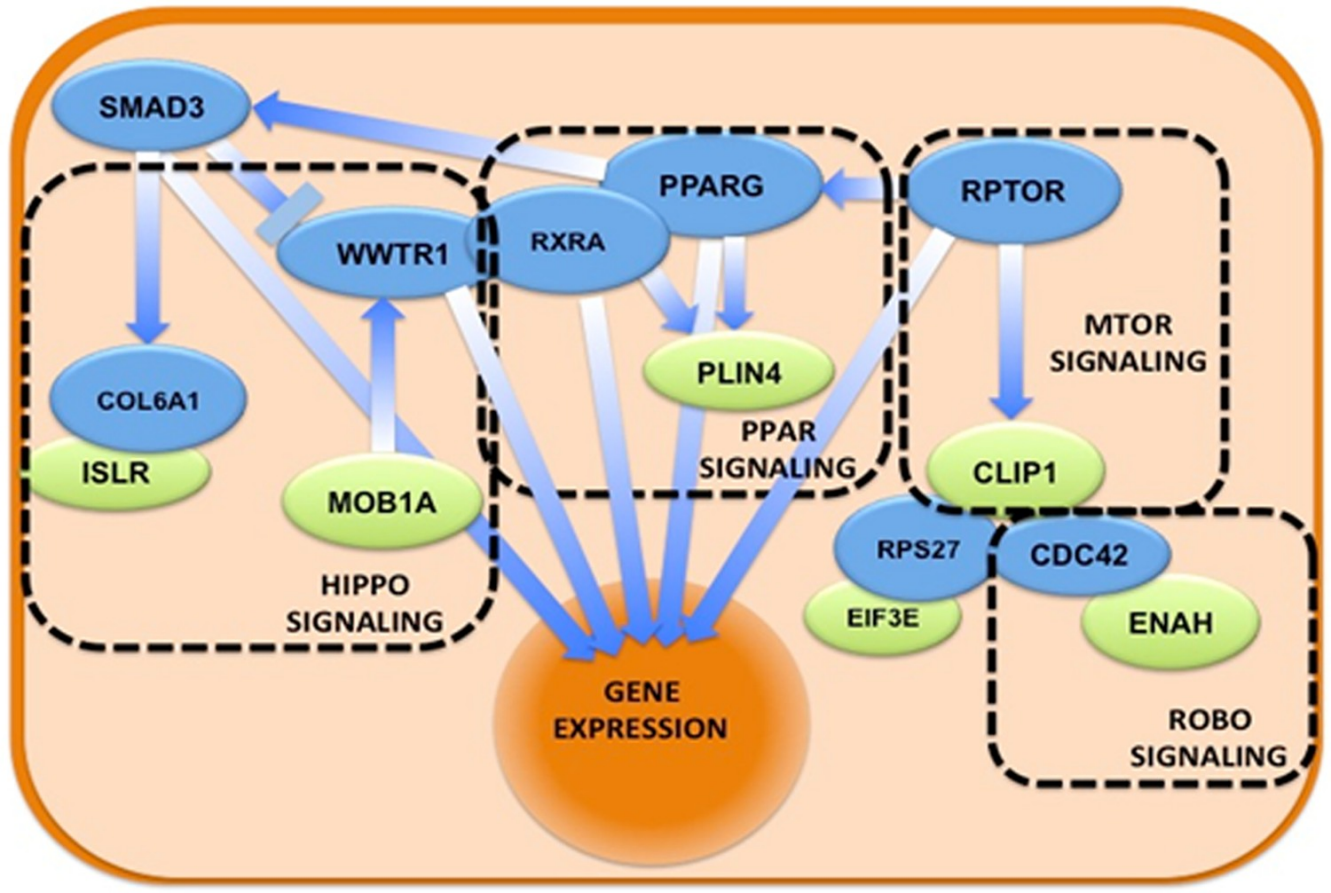
The biological network obtained using linker nodes for stage 2 breast cancer samples. Schematic representation of the molecular pathways in which the proteins of the set participate. Green ellipses represent proteins, while blue ellipses represent proteins that link all the input proteins. Overlapping proteins suggest physical interactions, while arrows indicate direct regulation. In dashed rectangles the different biological pathways that Reactome-FI detects as overrepresented in the full. The nucleus is represented as a wide orange ellipse because several pathways end with the effect on gene expression.

The full biological network obtained in Cytoscape (Fig 6) is notable in that the roles of linker nodes are filled by transcription factors, including RXRA, PPARG, RPTOR, WWTR1, and SMAD3, all of which are known to interact with one another and unify the input proteins involved in Hippo, Robo receptor, MTOR, and PPAR signaling pathways.

Our results clearly show alterations in multiple signaling pathways in stage 2 breast cancer, and the functions of the proteins involved are illustrated in Table 4. Notably, many impact proliferation, invasion, and migration pathways.

**Table 4:** List of protein functions identified in stage 2 breast cancer samples.

No.	Protein	Function	Reference
**1**	Prolyl 3-hydroxylase 1	Osteogenesis imperfecta	[39]
**2**	Peptidyl-prolyl cis-trans isomerase FKBP10	Osteogenesis imperfecta	[40]
**3**	CAP-Gly domain-containing linker protein 1	proliferation	[41]
**4**	Peptidyl-prolyl cis-trans isomerase FKBP9	Stress	[42]
**5**	Zinc finger CCCH domain-containing protein 18	Proliferation	[43]
**6**	Immunoglobulin superfamily containing leucine-rich repeat protein	Differentiation	[44]
**7**	MOB kinase activator 1A	proliferation	[45]
**8**	Protein enabled homolog	Tumor invasion	[46]
**9**	Collagen alpha-1 (V) chain	Pro-metastatic	[47]
**10**	Protein canopy homolog 4	Pro-metastatic	[47]
**11**	Perilipin-4 (PLIN4)	Adipogenesis	[48]
**12**	Transmembrane emp24 domain-containing protein 10	Pro-metastatic	[49]

### Proteins pathway for stage 3 breast cancer

**Fig 7:**
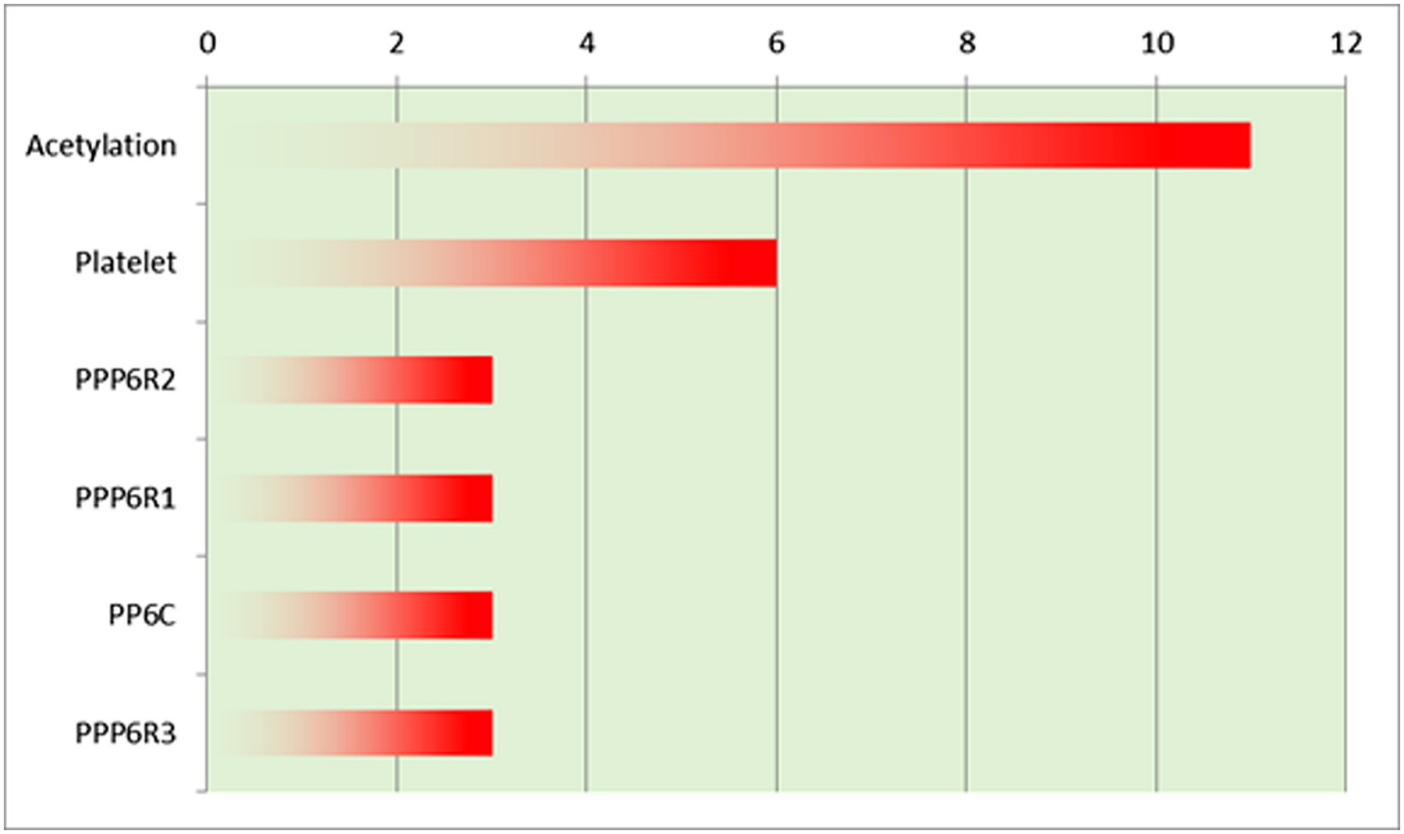
DAVID analysis on stage 3 breast cancer samples.

The analysis of this set by DAVID revealed a greater presence of proteins that can be acetylated, which suggests the involvement of a PTM in breast cancer progression to stage 3 (Fig 7 and S4 Table).

**Fig 8:**
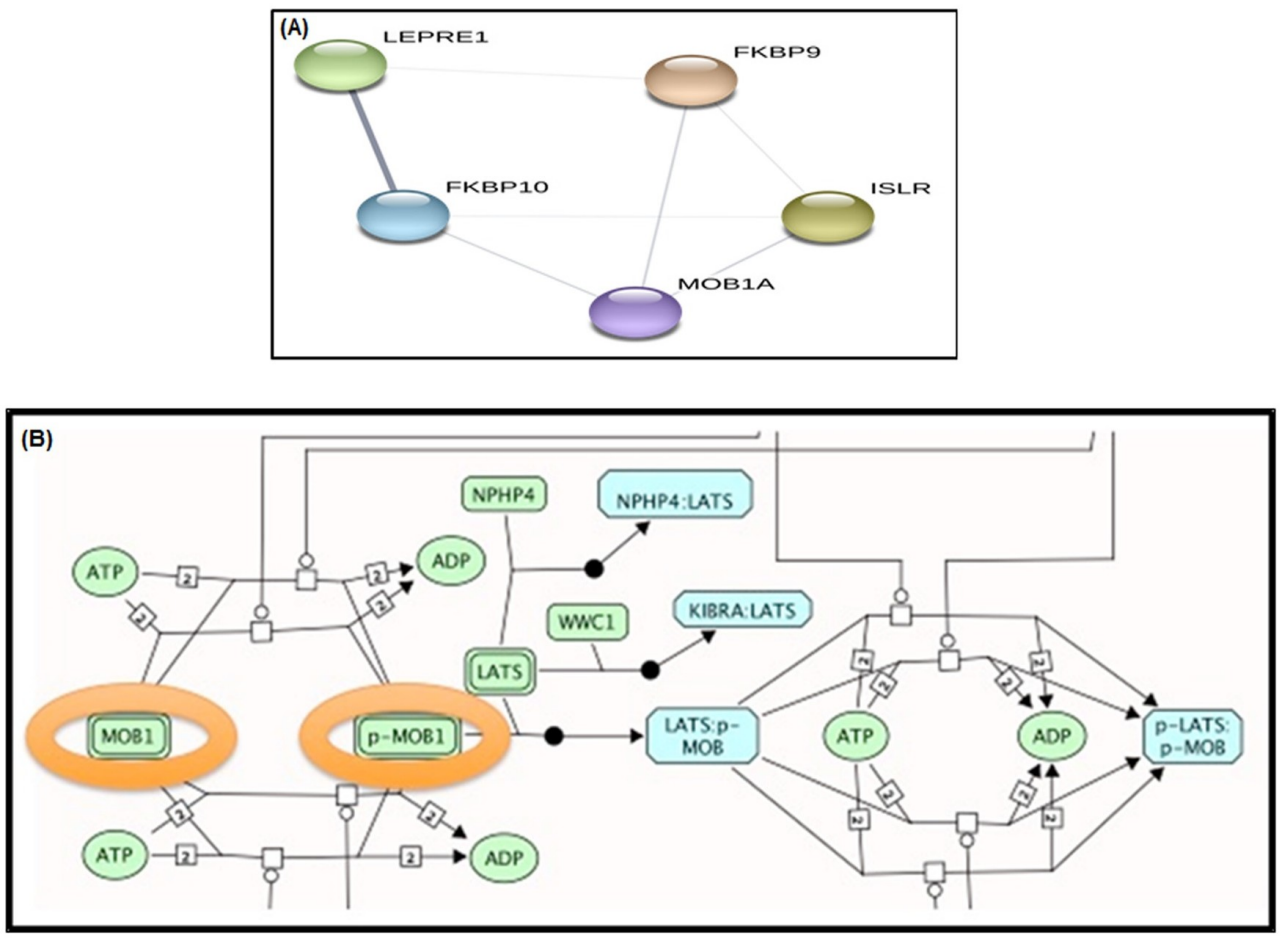
(A) Functional network of proteins in stage 3 breast cancer samples. (B) Molecular pathway of the proteins in stage 3 breast cancer samples, as predicted by STRING.

The STRING functional network shows in Fig 8A that there is a remarkable core node, SAMHD1 that is independently related to three different proteins and may represent a pathway regulator. It shows a non-directed graph with the functional interactions in the set as predicted by STRING. Each node represents a protein, Edges represent functional interaction between the proteins, so an edge between protein X and Y means that there is a predicted interaction between these proteins. This could represent a protein regulated by another, a genetic interaction, a physical interaction, the participation in the same biological process or the common regulation of both proteins by a third (known or unknown) one, among others. Nevertheless, these proteins are not present in a shared pathway. The other subnetwork among these proteins contains MOB1A and MOB1B, both of which represent the MOB1 protein, an important regulator of the Hippo signaling pathway that can be affected deeply by the presence of these proteins (Fig 8B).

**Fig 9:**
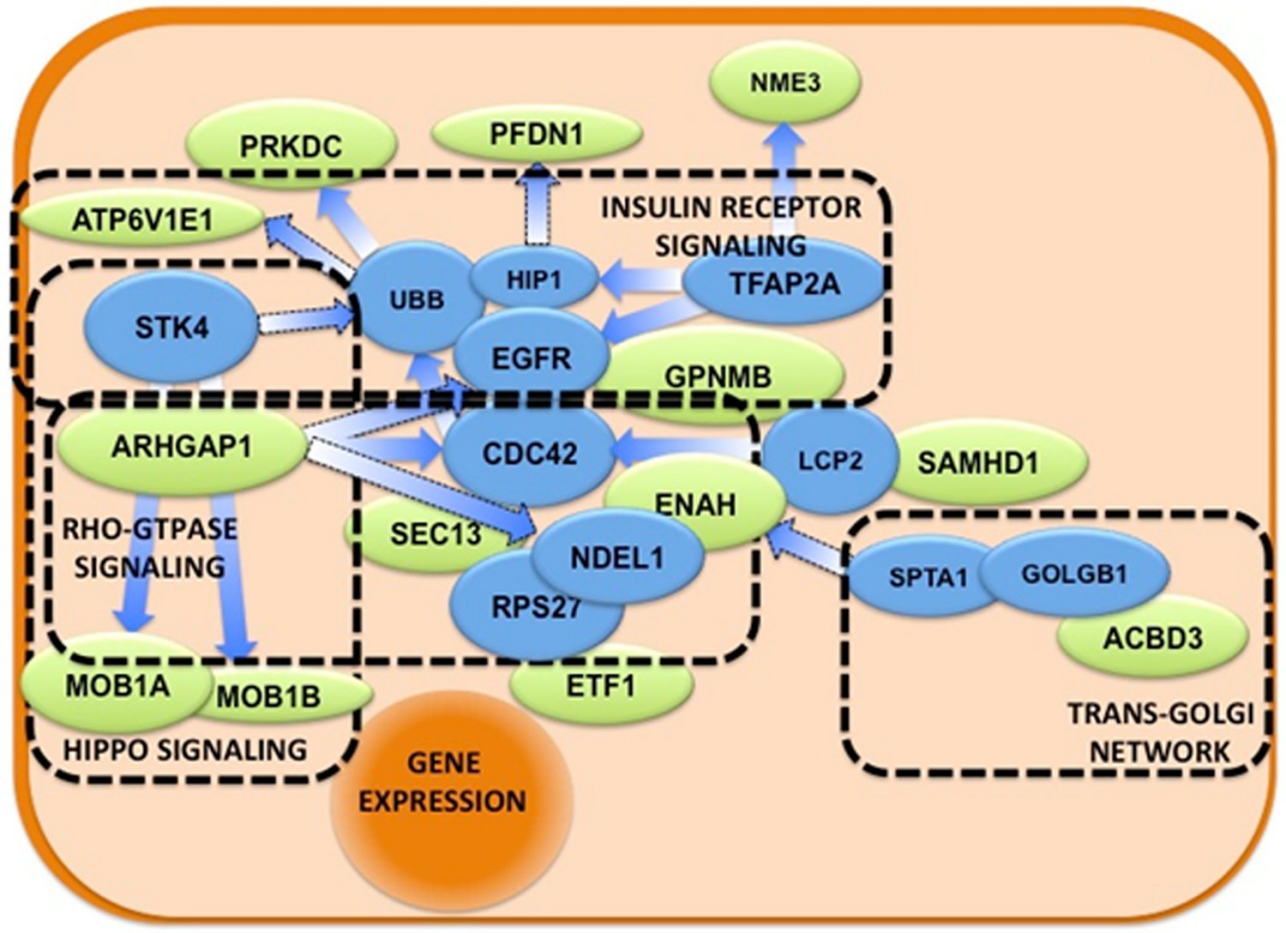
The biological network obtained using linker nodes for stage 3 breast cancer samples. Schematic representation of the molecular pathways in which the proteins of the set participate. Green ellipses represent proteins in the set, while blue ellipses represent ‘connector’ proteins that link all the input proteins. Overlapping proteins suggest physical interactions, while arrows indicate direct regulation. In dashed rectangles the different biological pathways that Reactome-FI detects as overrepresented in the full list (input proteins + connectors).

This figure shows a snapshot of a part of the Hippo regulatory pathway. The proteins from the list that participate in the process (MOB1A and B) are encircled, representing their role in Hippo pathway. Their phosphorylation (MOB1 to p-MOB1) is crucial for the final activation of the pathway. The biological network with linker nodes displays important cellular pathways like Hippo, Insulin Receptor, and Rho-GTPase signaling, as well as the trans-Golgi network (Fig 9). Two key proteins in cell physiology—CDC42 and EGFR—seem to be central to these pathways, suggesting their relevance for tumor progression to stage 3.

Thus, its relation to detected proteins in stage 3 breast cancer is remarkable. The functions of proteins detected in stage 3 are illustrated in Table 5. Interestingly, a subset of these proteins is related to metastasis, which supports the previous pathway results. The complete list of GO and other pathway terms enriched in the set among stage 3 breast cancer samples (S5 Table).

**Table 5:** List of proteins functions identified in stage 3 breast cancer samples.

No.	Protein Name	Function	Reference
**1**	Golgi resident protein GCP60	Stress	[50]
**2**	Eukaryotic peptide chain release factor subunit 1	Proliferation	[51]
**3**	Nucleoside diphosphate kinase 3	DNA repair	[52]
**4**	Deoxynucleosidetriphosphate triphosphohydrolase SAMHD1	Tumor suppression	[53]
**5**	Protein SEC13 homolog	Inflammation	[54]
**6**	Protein enabled homolog	Tumor invasion	[55]
**7**	Rho GTPase-activating protein 1	Anti-proliferation	[56]
**8**	LEM domain-containing protein 2	Differentiation	[57]
**9**	TAR DNA-binding protein 43 (Fragment)	Glycolysis	[58]
**10**	V-type proton ATPase subunit E 1	Metastasis	[59]
**11**	Prefoldin subunit 1	Pro-metastasis	[55]
**12**	Coiled-coil domain-containing protein 58 (Fragment)	Resistance to infection	[60]
**13**	Inhibitor of nuclear factor kappa-B kinase-interacting protein	Stress	[61]
**14**	DNA-dependent protein kinase catalytic subunit	Proliferation	[62]
**15**	Transmembrane glycoprotein NMB	Proliferation	[63]
**16**	MOB kinase activator 1A	Proliferation	[45]
**17**	MOB kinase activator 1B	Proliferation	[45]

## Discussion

In this work, we compared the proteomics profiling on samples of both breast cancer and adjacent normal tissues using advanced techniques using LTQ-Orbitrap, the GELFREE fraction system, and bioinformatics PEAKS software. Clinical proteomics remains the tool of choice for biochemical studies of cancer since it measures gene end products (proteins) to identify biomarkers in breast tissues. Therefore, identification of protein biomarkers may be employed in the diagnosis and/or treatment of breast cancer as useful biomarkers of biological function. Breast cancer is known as the most common type of cancers leading cause of death in women. Here, while this study founded an up-regulation in the expression of prolyl 3-hydroxylase 1 in stage 2 of breast cancer tissue compared with normal tissue, [64] reported the down-regulation of this protein. This protein is required for normal bone formation, and a deficiency in this protein causes a similar effect as the bone disorder, osteogenesis imperfecta [39].

Another probable mediator of the osteogenesis imperfect pathway is Peptidyl-prolyl cis-trans isomerase FKBP10 [40]. Our result showed FKBP10 was up-regulated in stage 2 breast cancer tissue, aligning with the finding of Ge et al. [65] that FKBP10 is overexpressed in renal cell carcinoma. In addition, it is suggested that FKBP10 could be a new promising therapeutic target for the treatment of renal cell carcinoma. These results imply that FKBP10 could be a promising biomarker and therapeutic option for breast cancer.

A related protein, FKBP9, also was up-regulated in stage 2 breast cancer tissue. This entire family of isomerases are named for their ability to bind immunosuppressive drugs and are responsible for the catalysis of the cis-trans conversion of peptidyl-prolyl bonds [66]. Previous studies have identified 16 human FKBP proteins ranging from 12 to 135 kDa, and have suggested that this specific immunophilin plays a critical role in tumorigenesis [65]. CAP-Gly domain-containing linker protein 1 was up-regulated in stage 2 breast cancer tissues and is known to be involved in proliferation [41].

Another novel finding of this study is the significant up-regulation of zinc finger CCCH domain-containing protein 18 in the tumor tissue sample compared with the normal tissue sample. This protein belongs to a protein family designated as MCPIP1, 2, 3, and 4 (encoded by the genes Zc3h12a–d). Although a distinctive feature of this protein family is the single CCCH-type zinc finger, we do not yet understand the actual function of these proteins as a whole [67], but protein 18 in the family is involved in proliferation [43].

Receptor tyrosine kinases can be inhibited by various tumor suppressors, most notably leucine-rich repeats and immunoglobulin-like domains 1 (LRIG1). It still not well understood how LRIG1 actually suppresses the activity of receptor tyrosine kinases on a molecular level [68]. The immunoglobulin superfamily contains leucine-rich repeats that are highly concentrated in stage 2 breast cancer samples. The function seems to be important for differentiation, protein-protein interactions, and cell adhesion, so it has been speculated that this class of proteins may interact with other proteins or cells [44].

In this study, MOB kinase activator 1A (MOB1A) was detected in stage 2, while both MOB1A and MOB1B were detected in stage 3 breast cancer samples. These findings are similar to what has been reported by Shen et al. [69], which revealed that MOB1A was up-regulated in breast cancer tissue. Furthermore, Shen et al. [69] explained that the roles of MOB kinase activator 1A include activation of LATS1/2 in the Hippo signaling pathway, restriction of proliferation, and promotion of apoptosis. Interestingly, other studies have shown that MOB1A can restrict the growth of cancer by activating the tumor-suppressing Hippo signaling pathway; this actually induced apoptosis in several cancer cell lines [70]. Nishio et al. [71] have established that the functions of MOB1A and MOB1B in skin homeostasis clearly overlap. They also explained that these two proteins can function as tumor suppressors specifically when they are downstream in the Hippo pathway.

Recent studies show overexpression of protein enabled homolog (ENAH) in several cancer types, and it has been shown to correlates with tumor invasiveness [72]. This study detected ENAH in both stages 2 and 3 and found evidence that it is important in cellular signaling. It is involved in carcinogenesis because certain invasive behaviors of breast cancer cells depend on the enzyme PI3K, induced by platelet-derived growth factor [73]. With this invasive trait, breast cancer cells can migrate and even gain metastatic potential [74].

Collagen type V performs a regulatory function and is also known to be up-regulated in multiple types of malignant tumors [75]. For example, it is expressed in the stroma of pancreatic ductal adenocarcinoma, where it is known to affect cell-cell adhesion, migration, and viability [47]. These functions do not seem to be impacted significantly even by chemotherapeutic drugs. Other studies have concurred, noting that collagen type V is up-regulated in breast cancer and colon cancer [76, 77]. Collagen type V is usually found in the extracellular matrix, where it influences tissue development, but it has also been discovered in association with cancers [78]. When breast cancer tissue becomes inflamed, collagen V is produced by adipocytes and macrophages. It increases resistance to chemotherapy and may therefore be a useful biomarker for cancer diagnosis. This study detected collagen type V in stage 2 breast cancer tissue. This finding bolsters evidence for this molecule’s role in tumor progression and alludes to the potential usefulness of collagen VI as a prognostic factor in the treatment of breast cancer [79].

In a novel departure from other studies, we found an up-regulation in the expressions of both canopy homolog 4 and perilipin (PLIN4) in stage 2 breast cancer tissues. Satish et al. [48] reported that PLIN4 is overexpressed in cancer and is a known marker for differentiated adipocytes since PLIN4 is involved in adipogenesis. Transmembrane emp24 domain-containing protein 10 (TMED10) was detected in stage 2 breast cancer tissues, concurring with Dong et al. [80]. They used a similar proteomics approach and reported TMED10 among the 13 unique proteins in oral squamous cell carcinoma. Golgi resident protein GCP60 (GOCAP1) was detected in the protein profile of stage 3 breast cancer samples. Fan et al. [81] reported that GOCAP1 protein can interact with golgin-160 fragments to regulate cell apoptosis.

We also detected Nucleoside diphosphate kinase 3(NDKA) in stage 3 breast cancer sample, aligning with the finding of Otero-Estévez and collaborators that a decrease in NDKA protein and mRNA has been associated with an increase in metastatic potential and poor prognosis in breast cancer. According to Yokdang et al. [82], a high level of NDKA may constitute both a biomarker and a therapeutic target in the management of breast cancer.

The current study detected Rho GTPase-activating protein 1 in stage 3 breast cancer tissues, in agreement with Wang et al. [72] and Burbelo et al. [83]. The Rho GTPase-activating protein 1 regulates both cytokinesis and cell differentiation. Wang et al. [72] revealed that previous studies had not fully considered the oncogenic role of Rho GTPase-activating protein 1 in gastric cancer, colorectal cancer, and breast cancer. Furthermore, previous studies have shown that both the growth and metastasis of breast cancer cells are affected by Rho GTPase signaling pathways. Rho GTPase protein levels, activation states, and effector protein abundances undergo alterations in breast cancer, and in some patients, these alterations ultimately promote cell growth, invasion, and metastasis [83].

This study detected LEM domain-containing protein 2 in stage 3 breast cancer tissues. This finding is consistent with Sasahira et al. [84], however, they evaluated LEM domain-containing protein 2 in the context of oral squamous cell carcinoma tumorigenesis and found that it may be important molecular marker of that cancer [84].

TAR DNA-binding protein 43 (TDP43) was detected in stage 3 breast cancer samples. This was a somewhat novel finding because previous studies have been less conclusive on the up-regulation, or lack thereof, of TDP43 in malignant tumors. For example, it has been found to be up-regulated in melanoma [85] and hepatocellular carcinoma [58]. Surprisingly, one study found that the presence of TDP43 actually indicated a good prognosis in neuroblastoma and breast cancer [86]. Another study found that in breast cancer specifically, a knockdown of TDP43 slowed tumor progression by interfering with cellular proliferation and metastatic potential, while overexpression promoted the proliferation of mammary epithelial cells [87]. The latter finding aligns with the results of the present study.

This study detected V-type proton ATPase subunit E 1 protein in stage 3 breast cancer tissues. This protein has a well-established role in metastasis [59]. Coiled-coil domain-containing protein 58 was detected in stage 3 breast cancer tissues. Gong et al. [88] found a direct link between the elevated expression of the coiled-coil domain family of proteins and the outcomes of tumor cell migration, invasion, and metastasis. Remarkably, this link was demonstrated in many cancers including those of the nasopharynx, stomach, prostate, pancreas, breast, and colon. The MTOR signaling pathway is very relevant in breast cancer [89] due to its control of cell growth and metabolic state. Robo is another pathway directly implicated in breast cancer [90].

Stage 3 breast cancer samples contained the inhibitor of nuclear factor kappa-B (NF-kB) kinase-interacting protein (AKIP1). This protein has been identified in breast cancer tissues previously by Kitching, Li [91], and the particular expression level of AKIP1 seems to affect the NF-kB cascade by regulating the mode of PKA signaling. In addition, these results lend credence to the mediating role of AKIP1 in cancer progression [92].

DNA-dependent protein kinase catalytic subunit (DNA-PK) was detected in stage 3 breast cancer tissues. The down-regulation of DNA-PK seems to confer an increased risk of certain cancers [93], so prior studies have suggested that DNA-PK may suppress carcinogenesis [94]. Lee et al. [94] demonstrated the link between suppressed DNA-PKcs expression and the formation of stage 1 gastric cancer in humans.

Prefoldin (PFDN) subunit 1 was detected in stage 3 breast cancer tissue, in agreement with [46]. Previous studies have illustrated that PFDN is a co-chaperone protein that is widely accepted to have important roles in the folding of actin and tubulin monomers during cytoskeletal assembly. Wang et al. [46], revealed that for lung cancer specifically, the transforming growth factor/PFDN subunit 1/cyclin A axis is important for induction and metastasis.

Transmembrane glycoprotein NMB (GPNMB) was detected in stage 3 breast cancer tissues. This finding supports Tajima et al. [95], Maric et al. [96], and Rose et al. [97], among other studies with similar findings in malignancies including melanoma, glioma, breast cancer, and gastric cancer. On a physiological level, GPNMB improves cell invasion and motility, enabling metastasis. It is therefore logical that, according to Tajima et al. [95], a high level of GPNMB expression indicates a worse prognosis. This means that GPNMB should be recognized as an important candidate for targeted therapy of malignancies [96]. Rose et al. [97] showed that the expression of GPNMB is considerably elevated in the aggressive bone-metastatic subpopulations of 4T1 breast cancer cells. It is worth noting that the current study suggests that the up-regulation of GPNMB is associated with a less favorable prognosis, so GPNMB could be used as a prognostic marker for epithelial ovarian cancer (EOC) patients.

Efficient protein folding and degradation are critical processes in cancer cells, so it is unsurprisingly that several tumors have been observed to over-express protein chaperones [98]. In the case of breast cancer, BiP is an overexpressed chaperone that has become the main target for new drugs [98]. These results are consistent with other enriched gene ontology terms (GO) that are to ER secretion and are similarly related to precise protein folding and degradation.

Interestingly, the control of protein acetylation by ACC1 was found recently to be linked to breast cancer progression [99]. Our result strongly supported these preliminary findings by confirming that these proteins appeared in stage 3 breast cancer tissue. In addition, these proteins are known to be concentrated in the platelets, and platelet counts have also been linked to breast cancer prognosis [100], showing again that the data obtained in Stage 3 is directly related to progression or invasiveness of the malignancy. Rho GTPases like CDC42 are crucial effectors in cancer proliferation, as reviewed in Tang et al. [101]. EGFR is one of the main molecules altered in cancer [102], and a specific antibody-based drug, Gefitinib, has been approved for its use in breast cancer [103].

Gelsolin, Protein Daple, Heat shock protein HSP 90-beta, Alpha-1-antitrypsin, and Cathepsin D constitute serum biomarker signature for diagnosing early grade breast cancer. The panel of proteins also provides crucial information for a better understanding of molecular mechanisms underlying the inceptive stage of breast cancer [104]. Recently, Gomig et al. [105] showed a high similarity in the proteomic profile between contralateral and adjacent non-tumor breast tissues. Differences between the proteome of the malignant and non-tumor tissue groups of the same patients were identified, providing crucial insights into signaling pathways of the biological functions including the comprehensive protein networks of breast cancer progression. These proteins can be considered as potential therapeutic targets against tumor development and metastatic progression in breast cancer disease. In addition, Fang et al. [106] have also shown that the levels of exosomal HER2 expression were similar to those detected in tumor tissues. The microfluidic chip could constitute a new platform for breast cancer diagnosis and molecular classification.

In this study, we have analyzed the pathways with potential involvement in the proteomic alterations that were evident in our breast cancer samples. Molecular experiments should be conducted to check the computational predictions, but our preliminary results still are interesting to discuss. Stage 2 and stage 3 proteins contained significant exosome components, suggesting the relevance of these extracellular vesicles in breast cancer tissue. Future studies can clarify the functional overlap between these sets. Is there a common upstream pathway that controls the different sets of molecules, or are there independent mechanisms converging on exosomes?

Finally, the network structure suggests an important role for proteins, like PGK1, that are central pathway nodes (“hubs”) in which signaling may converge. Given its relevance in several types of metastatic cancer [34, 107], PGK1 was confirmed as a potential target by our proteomic experiments. Integrin signaling and focal adhesions are important cellular mechanisms that are prevalent in our biological networks with linker nodes. Furthermore, the Hippo signaling pathway clearly performs important mechanisms in cancer [108], though the nature of these mechanisms are not yet clear. Finally, some transcription factors (present mainly in protein lists) have such high relevance that they may regulate the gene expression of several of the proteins at the same time, indicating an upstream mechanism controlling the pathway.

## Conclusion

In this study, we have successfully identified protein profiles of breast cancer tissue in stages 2 and 3 breast cancer patients, with a high percentage of coverage, using high-resolution and high mass accuracy MS analysis. Some proteins were common in both stages and some were unique to either stage. Beyond protein identification, this study proposed interactions, functions, networks, protein signaling, and protein pathways for each profiled protein, in stage 2 and stage 3. This study is foundational for establishing a baseline understanding of these paths, which now can be clarified with further differential proteomics profiling of breast cancer and other cancer for carcinogenesis, and biomarker discovery for breast cancer.

Taken together, these results suggest that alterations in protein expression in breast cancer offer clues that can help researchers develop novel proteins, design strategies for screening and prevention, and target therapy more effectively. The insights that this study has contributed toward proteomics identification will advances efforts, across many types of cancers, to establish differential protein profiles in carcinogenesis. We hope that the results of this study will be validated for early breast cancer diagnoses and therapeutic targeting in the future. Future research should attempt to verify the presence and roles of these proteins and validate them as early biomarkers that may be useful in breast cancer diagnosis. Data of the present study provides an improved understanding of the signalling pathways that are implicated in breast cancer. Furthermore, we found sufficient involvement of the Hippo signaling pathway that we recommend additional analysis of its mechanism in carcinogenesis. Therefore, it can be concluded that the dysregulated proteins or the dysregulated pathways can be exploited as biomarkers or targets, respectively, to invent novel and effective therapeutic systems.

## Supporting information

S1 Table Fractionation conditions of the GELFREE 10% Mass Cartridge Kit. (PDF)

S2 Table List of proteins identified by LC-MS/MS, in stage 2 of breast cancer tissue and their details as shown in PEAKS software. (PDF)

S3 Table List of GO and other pathway terms enriched among stage 2 breast cancer samples, with their counts and P-values following DAVID. (PDF)

S4 Table A list of proteins identified by LC-MS/MS, in stage 3 of breast cancer tissue and their details as shown in PEAKS software. (PDF)

S5 Table List of GO and other pathway terms enriched in the set among stage 3 breast cancer samples, with their counts and P-values following DAVID. (PDF)

S1 Appendix Fractionation conditions of the GELFREE. (PDF)

S2 Appendix Uncropped and full images of SDS-PAGE for the GELFREE images. (PDF)

S3 Appendix Whole list of proteins identified by LC-MS/MS in stage 2 of breast cancer tissues. (CSV)

S4 Appendix Proteins score distributions and Venn diagrams. (PDF)

S5 Appendix Whole list of proteins identified by LC-MS/MS in stage 3 of breast cancer tissues. (CSV)
