# Supplementary material for: Comparative proteomic analysis of different stages of breast cancer tissues using ultra high performance liquid chromatography tandem mass spectrometer
Source: PLoS One. 2020 Jan 16;15(1):e0227404. doi: 10.1371/journal.pone.0227404 (PMC6964830; doi:10.1371/journal.pone.0227404)
Supplement: S1 Table — (PDF) [file pone.0227404.s001.pdf]

S1 Table. Fractionation conditions of the GELFREE 10% Mass Cartridge Kit

**Step**	**1**	**2**	**3**	**4**	**5**	**6**	**7**	**8**	**9**	**10**	**11**	**12**	**13**
**Voltage (V)**	50	50	50	50	50	100	100	100	100	100	100	100	100
**Fraction interval (min)**	16	36	2	3	4	3	4	5	7	10	15	20	35
**Total elapsed time (min)**	16	52	54	57	61	64	68	73	80	90	105	125	160
**Fraction No.**	---	1	2	3	4	5	6	7	8	9	10	11	12
