# Supplementary material for: Comparative proteomic analysis of different stages of breast cancer tissues using ultra high performance liquid chromatography tandem mass spectrometer
Source: PLoS One. 2020 Jan 16;15(1):e0227404. doi: 10.1371/journal.pone.0227404 (PMC6964830; doi:10.1371/journal.pone.0227404)
Supplement: S2 Table — (PDF) [file pone.0227404.s002.pdf]

S2 Table. List of proteins identified by LC-MS/MS, in stage 2 of breast cancer tissue and their details as shown in PEAKS Software.

Protein name	Protein ID	Accession	-10lgP	Cov. (%)	#Peptides	#Unique	PTM	Avg. Mass	Description
**Prolyl 3-hydroxylase 1**	22919	sp|Q32P28|P3H1_HUMAN	147.87	25	10	10	Carbamidomethylation	83394	Prolyl 3-hydroxylase 1 OS=Homo sapiens GN=P3H1 PE=1 SV=2
**Peptidyl-prolyl cis-trans isomerase FKBP10**	22947	sp|Q96AY3|FKB10_HUMAN	149.64	30	2	1		64245	Peptidyl-prolyl cis-trans isomerase FKBP10 OS=Homo sapiens GN=FKBP10 PE=1 SV=1
**CAP-Gly domain-containing linker protein 1**	23712	sp|P30622|CLIP1_HUMAN	79.66	19	9	3	Carbamidomethylation; Oxidation (M)	162246	CAP-Gly domain-containing linker protein 1 OS=Homo sapiens GN=CLIP1 PE=1 SV=2
**Peptidyl-prolyl cis-trans isomerase FKBP9**	24130	sp|O95302|FKBP9_HUMAN	92.02	18	4	4	Carbamidomethylation	63084	Peptidyl-prolyl cis-trans isomerase FKBP9 OS=Homo sapiens GN=FKBP9 PE=1 SV=2
**Zinc finger CCCH domain-containing protein 18**	24279	sp|Q86VM9|ZCH18_HUMAN	69.13	15	3	3	Carbamidomethylation	106379	Zinc finger CCCH domain-containing protein 18 OS=Homo sapiens GN=ZC3H18 PE=1 SV=2
**Immunoglobulin superfamily containing leucine-rich repeat protein**	24971	sp|O14498|ISLR_HUMAN	67.06	15	3	3		45997	Immunoglobulin superfamily containing leucine-rich repeat protein OS=Homo sapiens GN=ISLR PE=2 SV=1
**MOB kinase activator 1A**	24983	sp|Q9H8S9|MOB1A_HUMAN	78.5	16	9	3	Carbamidomethylation; Oxidation (M)	25080	MOB kinase activator 1A OS=Homo sapiens GN=MOB1A PE=1 SV=4
**Protein enabled homolog**	22965	sp|Q8N8S7|ENAH_HUMAN	94.18	20	5	4		66510	Protein enabled homolog OS=Homo sapiens GN=ENAH PE=1 SV=2
**Collagen alpha-1(V) chain**	23027	tr|A0A087WXW9|A0A087WXW9_HUMAN	133.04	18	5	4	Carbamidomethylation	183652	Collagen alpha-1(V) chain OS=Homo sapiens GN=COL5A1 PE=1 SV=1
**Protein canopy homolog 4**	23031	tr|A0A087WXW9|A0A087WXW9_HUMAN	153.34	22	4	4	Carbamidomethylation	183652	Collagen alpha-1(V) chain OS=Homo sapiens GN=COL5A1 PE=1 SV=1
**Perilipin-4 (PLIN4)**	18	sp|Q96Q06|PLIN4_HUMAN	192.58	32	4	2	Carbamidomethylation	134431	Perilipin-4 OS=Homo sapiens GN=PLIN4 PE=1 SV=2
**Transmembrane emp24 domain-containing protein 10**	3026	sp|P60228|EIF3E_HUMAN	114.05	24	4	4	Carbamidomethylation; Oxidation (M)	52221	Eukaryotic translation initiation factor 3 subunit E OS=Homo sapiens GN=EIF3E PE=1 SV=1
