# Supplementary material for: Comparative proteomic analysis of different stages of breast cancer tissues using ultra high performance liquid chromatography tandem mass spectrometer
Source: PLoS One. 2020 Jan 16;15(1):e0227404. doi: 10.1371/journal.pone.0227404 (PMC6964830; doi:10.1371/journal.pone.0227404)
Supplement: S3 Table — (PDF) [file pone.0227404.s003.pdf]

S3 Table. List of GO and other pathway terms enriched among stage 2 breast cancer samples, with their counts and P-values following DAVID.

Term	Count	P-Value
GO:0061077~chaperone-mediated protein folding	3	1,31E+12
short sequence motif:Prevents secretion from ER	3	4,03E+11
domain:PPIase FKBP-type 4	2	9,97E+10
domain:PPIase FKBP-type 3	2	9,97E+10
domain:PPIase FKBP-type 1	2	0.002
domain:PPIase FKBP-type 2	2	0.002
GO:0006457~protein folding	3	0.003
Metal-binding	7	0.003
HTF	10	0.004
GO:0005788~endoplasmic reticulum lumen	3	0.005
847:ovary_normal_3rd	3	0.006
8560:delta 4-desaturase, sphingolipid 1(DEGS1)	2	0.009
GO:0005528~FK506 binding	2	0.009
IPR001179:Peptidyl-prolyl cis-trans isomerase, FKBP-type, domain	2	0.010
Osteogenesis imperfect	2	0.010
IPR023566:Peptidyl-prolyl cis-trans isomerase, FKBP-type	2	0.011
13728:uncharacterized tissue_neoplasia_3rd	2	0.013
51400:PPME1~protein phosphatase methylesterase 1	2	0.017
Rotamase	2	0.018
11385:uncharacterized tissue_neoplasia_3rd	2	0.019
27173:thyroid_neoplasia_3rd	2	0.020
GO:0000413~protein peptidyl-prolyl isomerization	2	0.020
GO:0003755~peptidyl-prolyl cis-trans isomerase activity	2	0.022
23640:HSPBP1~HSPA (Hsp70) binding protein 1	2	0.022
P53	10	0.025
17518:thyroid_neoplasia_3rd	2	0.026
Teratocarcinoma	3	0.026
10956:colon_neoplasia_3rd	2	0.027
21686:brain_neoplasia_3rd	2	0.031

4898:NRDC~nardilysin convertase	2	0.035
8882:ZPR1~ZPR1 zinc finger	2	0.036
11187:head and neck_neoplasia_3rd	2	0.036
21717:placenta_normal_3rd	2	0.040
MEIS1	8	0.043
19240:head and neck_neoplasia_3rd	2	0.047
19945:uncharacterized tissue_normal_3rd	2	0.049
GRE	8	0.049
LMO2COM	9	0.050

In this table, we show the results from DAVID analysis using proteins for stage 2 samples set. The 'Term'is a name or the ID of a biochemical pathway/gene ontology term/tissue/protein interaction/transcription factor that is enriched in our set. This means that the number of proteins in our list that have been associated to this term (e.g., because they have a PPIase domain or they play a role in chaperone folding) are significantly higher than a background list (in this case, the whole genome). This significance is shown in the P-value column, that is the parameter by which we rank the results, having a P-value threshold of P=0.05. Finally, we also show the total number of proteins in our list that are represented by each term, in the Count column. This number is not directly correlated to the P-value (that would mean that higher count was related to lower P-values) because the number of proteins for each term is different in the genomic backgrpund (i.e., there are more genes in the genome associated to HTF transcription factor than to protein folding. because 10 proteins in our list are associated to HTF for a p-value of 0.004, while only 3 proteins in our list associated to protein folding produce a p-value of 10^-12
