# Supplementary material for: Comparative proteomic analysis of different stages of breast cancer tissues using ultra high performance liquid chromatography tandem mass spectrometer
Source: PLoS One. 2020 Jan 16;15(1):e0227404. doi: 10.1371/journal.pone.0227404 (PMC6964830; doi:10.1371/journal.pone.0227404)
Supplement: S4 Table — (PDF) [file pone.0227404.s004.pdf]

S4 Table. A list of proteins identified by LC-MS/MS, in stage 3 of breast cancer tissue and their details as shown in PEAKS Software.

Protein name	Protein ID	Accession	-10lgP	Cov. (%)	#Peptides	#Unique	PTM	Avg. Mass	Description
**Golgi resident protein GCP60**	23480	sp|Q9H3P7|GCP60_HUMAN	178.62	12	5	5	Carbamido methylation	60593	Golgi resident protein GCP60 OS=Homo sapiens GN=ACBD3 PE=1 SV=4
**Eukaryotic peptide chain release factor subunit 1**	2730	sp|P62495|ERF1_HUMAN	110.05	13	5	5	Carbamido methylation	49031	Eukaryotic peptide chain release factor subunit 1 OS=Homo sapiens GN=ETF1 PE=1 SV=3
**Nucleoside diphosphate kinase 3**	2753	sp|Q13232|NDK3_HUMAN	60	16	2	2	Carbamido methylation; Oxidation (M)	19015	Nucleoside diphosphate kinase 3 OS=Homo sapiens GN=NME3 PE=1 SV=2
**Deoxynucleoside triphosphate triphosphohydrolase SAMHD1**	3436	sp|Q9Y3Z3|SAMH1_HUMAN	111.21	21	10	10	Carbamido methylation	72201	Deoxynucleoside triphosphate triphosphohydrolase SAMHD1 OS=Homo sapiens GN=SAMHD1 PE=1 SV=2
**Protein SEC13 homolog**	3720	sp|P55735|SEC13_HUMAN	156.3	23	5	5	Carbamido methylation	35541	Protein SEC13 homolog OS=Homo sapiens GN=SEC13 PE=1 SV=3
**Protein SEC13 homolog**	3721	tr|A0A0C4DFR6|A0A0C4DFR6_HUMAN	43.91	16	3	3	Carbamido methylation	35857	Protein enabled homolog OS=Homo sapiens GN=ENAH PE=1 SV=2
**Protein enabled homolog**	22965	sp|Q8N8S7|ENAH_HUMAN	94.18	10	5	5	Carbamido methylation	66510	Rho GTPase-activating protein 1 OS=Homo sapiens GN=ARHGAP1 PE=1 SV=1
**Rho GTPase-activating protein 1**	23329	sp|Q07960|RHG01_HUMAN	124.29	15	7	7	Carbamido methylation; Oxidation (M)	50436	LEM domain-containing protein 2 OS=Homo sapiens GN=LEMD2 PE=1 SV=1
**LEM domain-containing protein 2**	23373	sp|Q8NC56|LEMD2_HUMAN	109.47	13	5	5	Carbamido methylation	56975	TAR DNA-binding protein 43 (Fragment) OS=Homo sapiens GN=TARDBP PE=1 SV=1
**TAR DNA-binding protein 43 (Fragment)**	23455	tr|A0A087WX29|A0A087WX29_HUMAN	82.04	18	4	4	Carbamido methylation	26743	V-type proton ATPase subunit E 1 OS=Homo sapiens GN=ATP6V1E1 PE=1 SV=1
**V-type proton ATPase subunit E 1**	23744	sp|P36543|VATE1_HUMAN	49.52	14	3	3	Carbamido methylation; Oxidation (M)	26145	Prefoldin subunit 1 OS=Homo sapiens GN=PFDN1 PE=1 SV=1
**Prefoldin subunit 1**	23751	sp|O60925|PFD1_HUMAN	64.03	24	3	3	Carbamido methylation; Oxidation (M)	14210	Coiled-coil domain-containing protein 58 (Fragment) OS=Homo sapiens GN=CCDC58 PE=1 SV=1
**Coiled-coil domain-containing protein 58 (Fragment)**	23932	tr|H7C525|H7C525_HUMAN	87.57	37	4	4	Carbamido methylation	16385	DNA-dependent protein kinase catalytic subunit OS=Homo sapiens GN=PRKDC PE=1 SV=3
**Inhibitor of nuclear factor kappa-B kinase-interacting protein**	24189	sp|Q70UQ0|IKIP_HUMAN	83.72	12	4	4	Carbamido methylation; Oxidation (M)	39309	Transmembrane glycoprotein NMB OS=Homo sapiens GN=GPNMB PE=1 SV=2
**DNA-dependent protein kinase**	24231	sp|P78527|PRKDC_HUMAN	114.44	12	12	12	Carbamido methylation	469093	MOB kinase activator 1A OS=Homo sapiens

**catalytic subunit**									GN=MOB1A PE=1 SV=4
**Transmembrane glycoprotein NMB**	24286	sp|Q14956|GPNMB_HUMAN	56.1	14	3	3	Carbamido methylation	63923	MOB kinase activator 1B OS=Homo sapiens GN=MOB1B PE=1 SV=3
**MOB kinase activator 1A**	24983	sp|Q9H8S9|MOB1A_HUMAN	73.49	18	8	3	Carbamido methylation	25080	Golgi resident protein GCP60 OS=Homo sapiens GN=ACBD3 PE=1 SV=4
**MOB kinase activator 1B**	24984	sp|Q7L9L4|MOB1B_HUMAN	73.49	19	2	2	Carbamido methylation;	25091	Eukaryotic peptide chain release factor subunit 1 OS=Homo sapiens GN=ETF1 PE=1 SV=3
