# Supplementary material for: Comparative proteomic analysis of different stages of breast cancer tissues using ultra high performance liquid chromatography tandem mass spectrometer
Source: PLoS One. 2020 Jan 16;15(1):e0227404. doi: 10.1371/journal.pone.0227404 (PMC6964830; doi:10.1371/journal.pone.0227404)
Supplement: S5 Table — (PDF) [file pone.0227404.s005.pdf]

S5 Table. List of GO and other pathway terms enriched in the set among stage 3 breast cancer samples, with their counts and P-values following DAVID.

Term	Count	P-Value
Acetylation	11	2,14E+10
Platelet	6	4,21E+10
9701:PPP6R2~protein phosphatase 6 regulatory subunit 2	3	1,67E+11
22870:PPP6R1~protein phosphatase 6 regulatory subunit 1	3	3,00E+11
5537:protein phosphatase 6 catalytic subunit(PPP6C)	3	3,41E+11
5537:PPP6C~protein phosphatase 6 catalytic subunit	3	4,98E+11
55291:PPP6R3~protein phosphatase 6 regulatory subunit 3	3	6,78E+11
FOXO4	14	0.001
39351:eye_uncharacterized histology_3rd	4	0.001
Phosphoprotein	13	0.002
6449:cervix_neoplasia_3rd	4	0.005
21381:lung_neoplasia_3rd	3	0.006
81532:MOB kinase activator 2(MOB2)	2	0.006
IPR005301:Mob1/phocein	2	0.006
9770:RASSF2~Ras association domain family member 2	2	0.006
5515:PPP2CA~protein phosphatase 2 catalytic subunit alpha	3	0.007
23143:leucine rich repeats and calponin homology domain containing 1(LRCH1)	2	0.007
1q42.12	2	0.008
51379:CRLF3~cytokine receptor like factor 3	2	0.008
81532:MOB2~MOB kinase activator 2	2	0.008
SM01388:SM01388	2	0.009
23143:LRCH1~leucine rich repeats and calponin homology domain containing 1	2	0.009
GO:0005515~protein binding	13	0.010
23012:serine/threonine kinase 38 like(STK38L)	2	0.011
26524:large tumor suppressor kinase 2(LATS2)	2	0.012
22872:SEC31 homolog A, COPII coat complex component(SEC31A)	2	0.012
57631:leucine rich repeats and calponin homology domain containing 2(LRCH2)	2	0.012
84859:LRCH3~leucine rich repeats and calponin homology domain containing 3	2	0.012
57631:LRCH2~leucine rich repeats and calponin homology domain containing 2	2	0.012
283373:ankyrin repeat domain 52(ANKRD52)	2	0.013
23012:STK38L~serine/threonine kinase 38 like	2	0.013
6789:serine/threonine kinase 4(STK4)	3	0.014
695:testi_neoplasia_3rd	3	0.014
51194:importin 11(IPO11)	2	0.014
5784:PTPN14~protein tyrosine phosphatase, non-receptor type 14	2	0.014
57572:DOCK6~dedicator of cytokinesis 6	2	0.016
57572:dedicator of cytokinesis 6(DOCK6)	2	0.016
283373:ANKRD52~ankyrin repeat domain 52	2	0.017
51379:cytokine receptor like factor 3(CRLF3)	2	0.017
11133:head and neck_neoplasia_3rd	2	0.017
60485:SAV1~salvador family WW domain containing protein 1	2	0.018

R-HSA-2028269:R-HSA-2028269	2	0.020
55291:protein phosphatase 6 regulatory subunit 3(PPP6R3)	2	0.020
988:cell division cycle 5 like(CDC5L)	4	0.020
81704:DOCK8~dedicator of cytokinesis 8	2	0.021
7247:TSN~translin	2	0.022
GO:0035329~hippo signaling	2	0.022
22938:SNW domain containing 1(SNW1)	4	0.023
84859:leucine rich repeats and calponin homology domain containing 3(LRCH3)	2	0.025
81704:dedicator of cytokinesis 8(DOCK8)	2	0.025
83593:RASSF5~Ras association domain family member 5	2	0.027
11124:head and neck_neoplasia_3rd	2	0.027
9701:protein phosphatase 6 regulatory subunit 2(PPP6R2)	2	0.027
351:APP~amyloid beta precursor protein	6	0.028
9770:Ras association domain family member 2(RASSF2)	2	0.028
20295:head and neck_neoplasia_3rd	2	0.032
6789:STK4~serine/threonine kinase 4	2	0.032
22872:SEC31A~SEC31 homolog A, COPII coat complex component	2	0.032
6232:mammary gland_neoplasia_3rd	2	0.032
26524:LATS2~large tumor suppressor kinase 2	2	0.033
38094:lung_uncharacterized histology_3rd	3	0.033
23243:ANKRD28~ankyrin repeat domain 28	2	0.034
51194:IPO11~importin 11	2	0.034
11329:STK38~serine/threonine kinase 38	2	0.035
17656:head and neck_neoplasia_3rd	2	0.035
85440:dedicator of cytokinesis 7(DOCK7)	2	0.036
29963:liver_neoplasia_3rd	3	0.036
6788:STK3~serine/threonine kinase 3	2	0.037
SRY	10	0.037
9113:LATS1~large tumor suppressor kinase 1	2	0.037
667:dystonin(DST)	2	0.038
11200:CHEK2~checkpoint kinase 2	2	0.038
83593:Ras association domain family member 5(RASSF5)	2	0.039
Cervix carcinoma	3	0.039
8894:EIF2S2~eukaryotic translation initiation factor 2 subunit beta	2	0.039
25929:gem nuclear organelle associated protein 5(GEMIN5)	2	0.040
EVI1	15	0.040
GO:0005829~cytosol	7	0.040
5784:protein tyrosine phosphatase, non-receptor type 14(PTPN14)	2	0.041
TBP	9	0.041
40211:uncharacterized tissue_uncharacterized histology_3rd	3	0.041
16556:uncharacterized tissue_uncharacterized histology_3rd	3	0.042
22870:protein phosphatase 6 regulatory subunit 1(PPP6R1)	2	0.043
SH3-binding	2	0.045
8663:EIF3C~eukaryotic translation initiation factor 3 subunit C	2	0.045
667:DST~dystonin	2	0.047
GO:0001933~negative regulation of protein phosphorylation	2	0.050

The 'Term' is a name or the ID of a biochemical pathway/gene ontology term/tissue/protein interaction/transcription factor that is enriched in our set. This means that the number of proteins in

our list that have been associated to this term (e.g., because they are expressed in platelets, they play a role in protein acetylation, or they interact with PPP6R2 or they are regulated by FOXO4 transcription factor) are significantly higher than a background list (in this case, the whole genome). This significance is shown in the P-value column, that is the parameter by which we rank the results, having a P-value threshold of P=0.05. Finally, we also show the total number of proteins in our list that are represented by each term, in the Count column. This number is not directly correlated to the P-value (that would mean that higher count was related to lower P-values) because the number of proteins for each term is different in the genomic background (i.e., there are more genes in the genome associated to FOXO4 transcription factor than to platelets. because 14 proteins in our list are associated to FOXO4 for a p-value of 0.001, while only 6 proteins in our list associated to platelets produce a p-value of 10^-10
