# Supplementary material for: Comparative proteomic analysis of different stages of breast cancer tissues using ultra high performance liquid chromatography tandem mass spectrometer
Source: PLoS One. 2020 Jan 16;15(1):e0227404. doi: 10.1371/journal.pone.0227404 (PMC6964830; doi:10.1371/journal.pone.0227404)
Supplement: S1 Appendix — (PDF) [file pone.0227404.s006.pdf]

GELFREE fractions

During our method development, fractionated GELFREE fractions were further analysed either separately or combined into four groups as listed in the Table A. The total protein concentration was also determined for each group. Then, 50 µg protein samples from each group were collected then frozen for 30 minutes at -80°C and then let freeze-dried overnight.

Table A. GELFREE fractions were combined into four groups

Group name	Fraction numbers
**Group 1**	Fractions 1-3
**Group 2**	Fractions 4-5
**Group 3**	Fractions 6-9
**Group 4**	Fractions 10-12
