# Supplementary figures and images for: Comparative proteomic analysis of different stages of breast cancer tissues using ultra high performance liquid chromatography tandem mass spectrometer
Source: PLoS One. 2020 Jan 16;15(1):e0227404. doi: 10.1371/journal.pone.0227404 (PMC6964830; doi:10.1371/journal.pone.0227404)

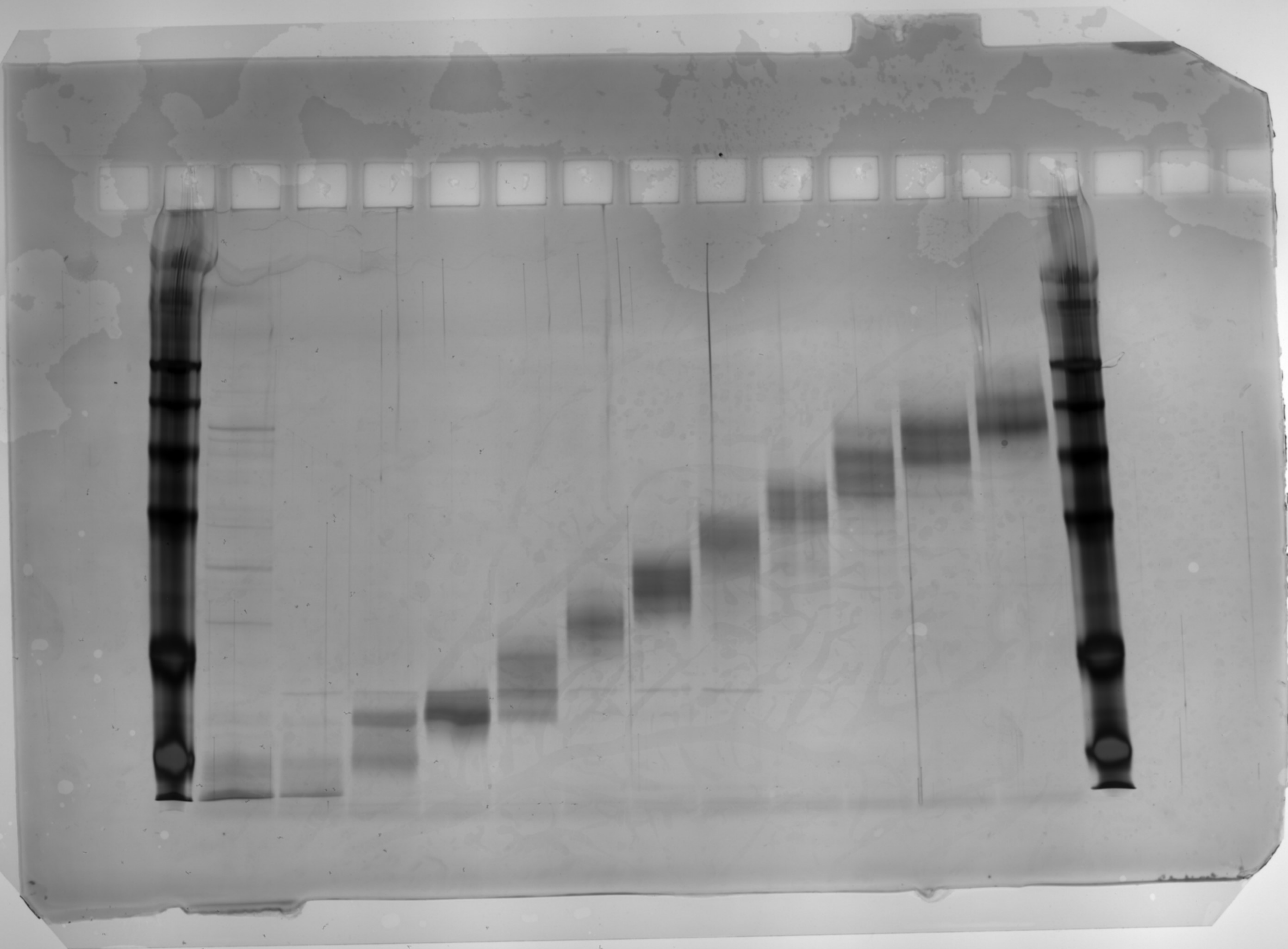

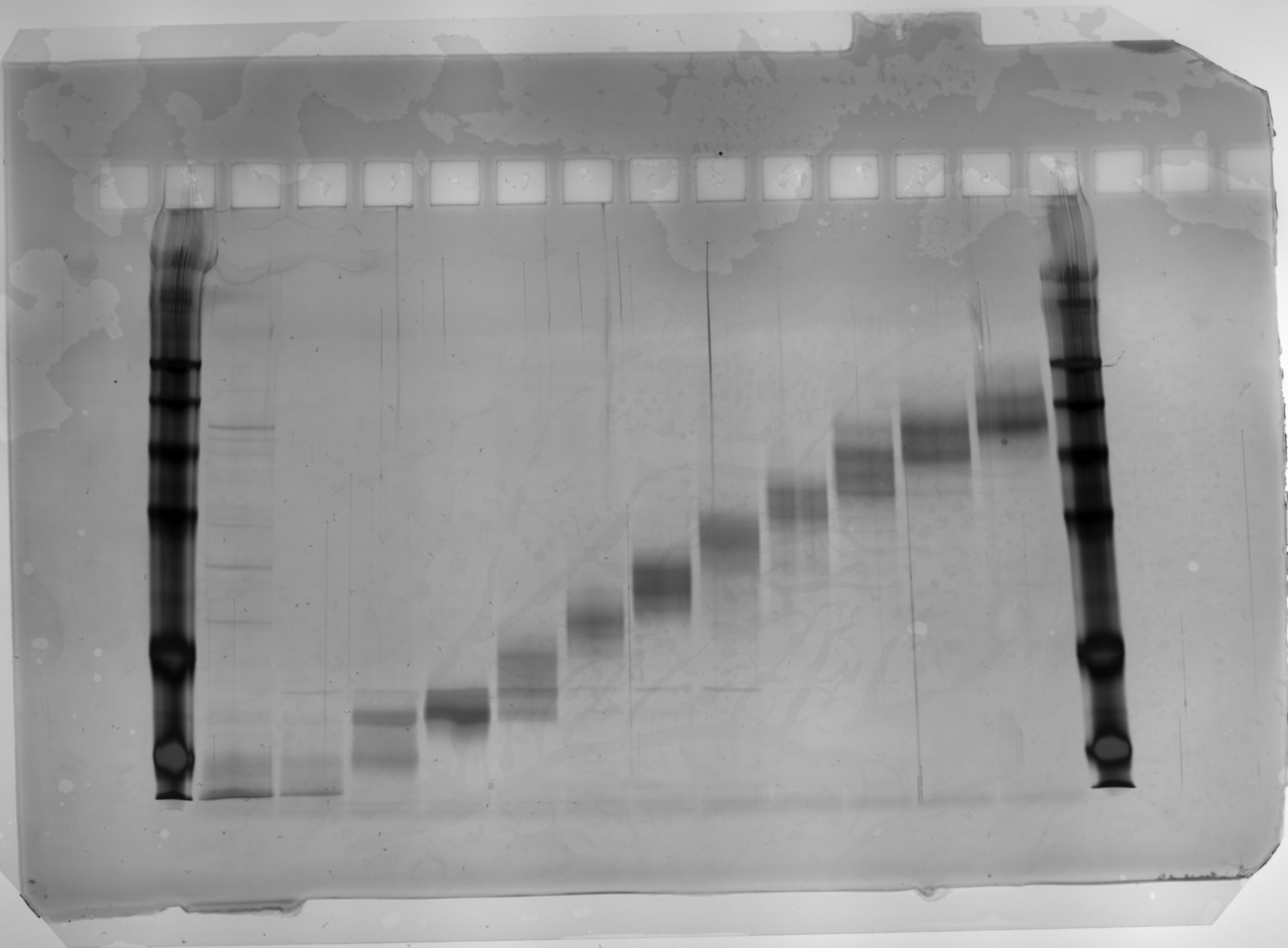

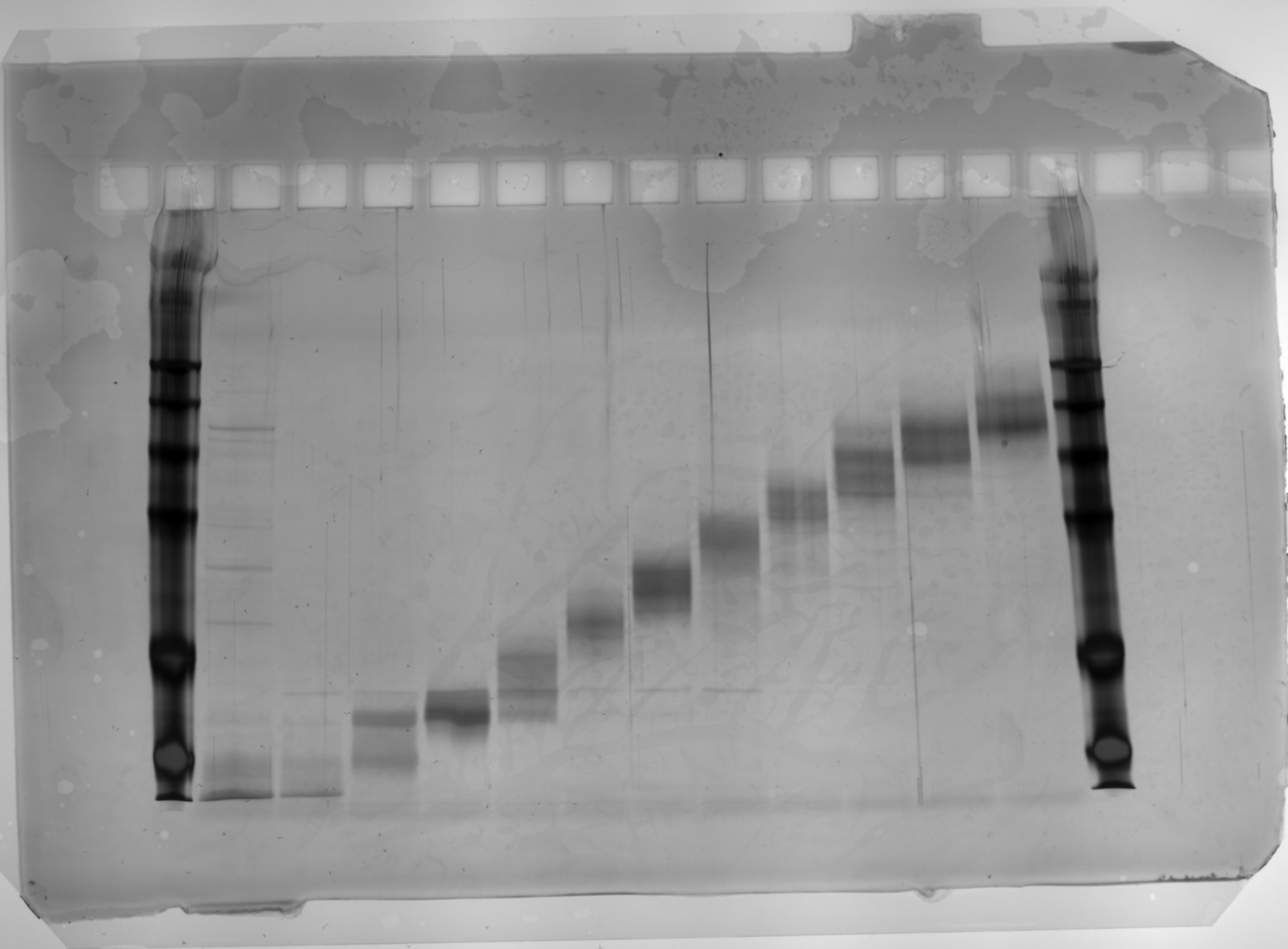

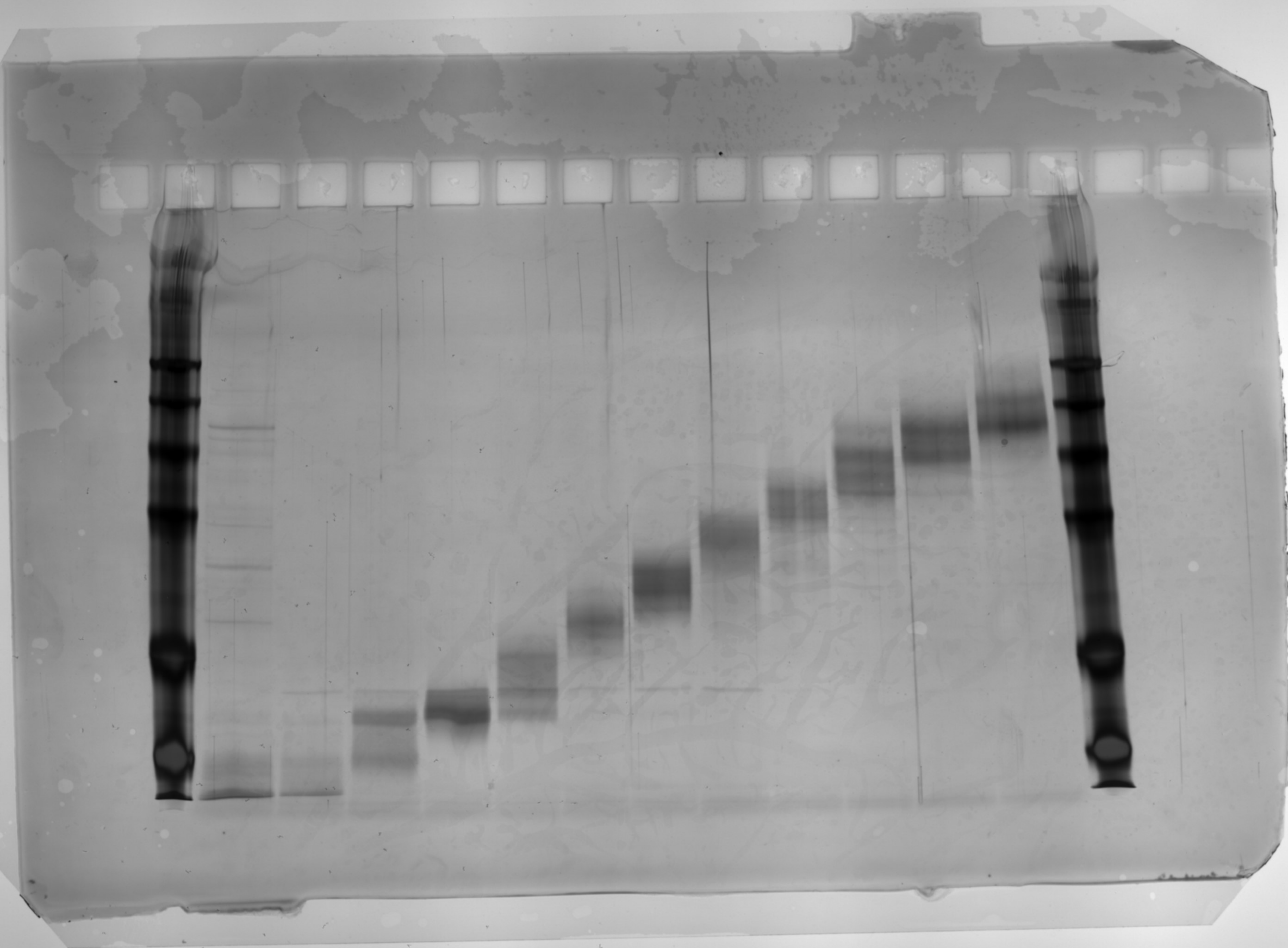

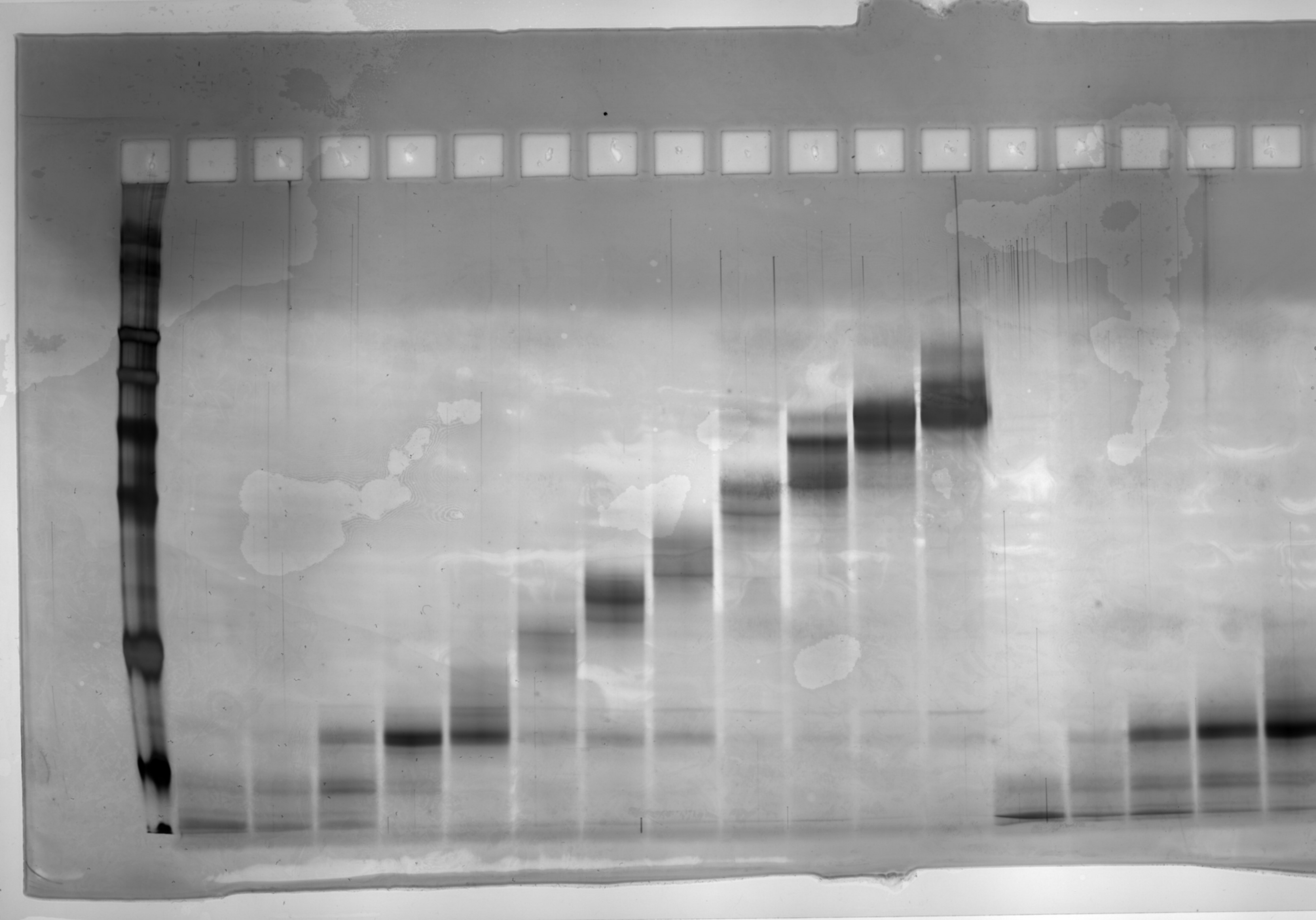

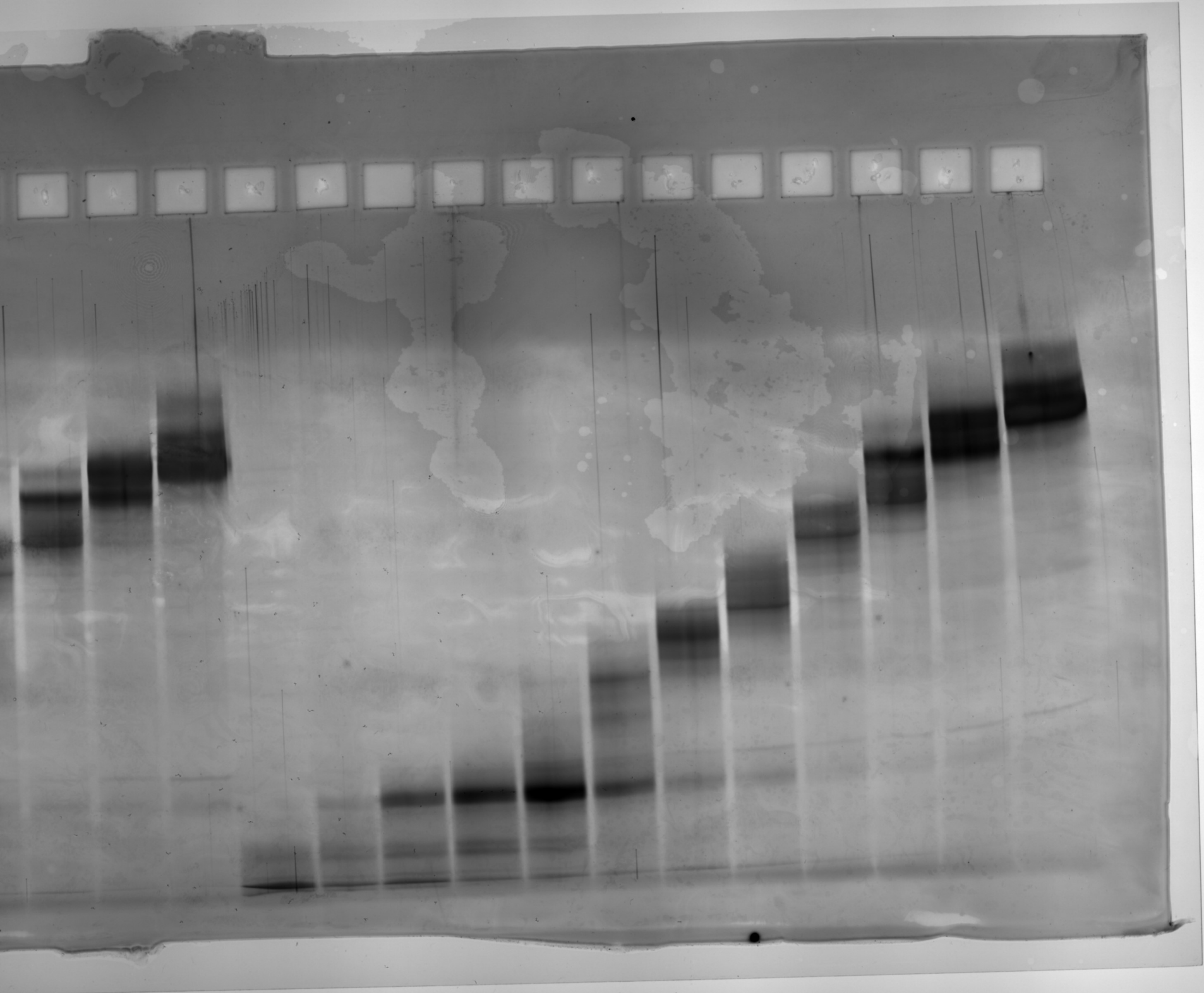

Supplement: S2 Appendix — (PDF) [file pone.0227404.s007.pdf]
