# Supplementary material for: Comparative proteomic analysis of different stages of breast cancer tissues using ultra high performance liquid chromatography tandem mass spectrometer
Source: PLoS One. 2020 Jan 16;15(1):e0227404. doi: 10.1371/journal.pone.0227404 (PMC6964830; doi:10.1371/journal.pone.0227404)
Supplement: S4 Appendix — (PDF) [file pone.0227404.s009.pdf]

<u>S5 Appendix.</u> PEAKS provides a number of statistical charts: peptide score distribution, peptide number Venn diagram, protein score distribution, and the protein number Venn diagram.

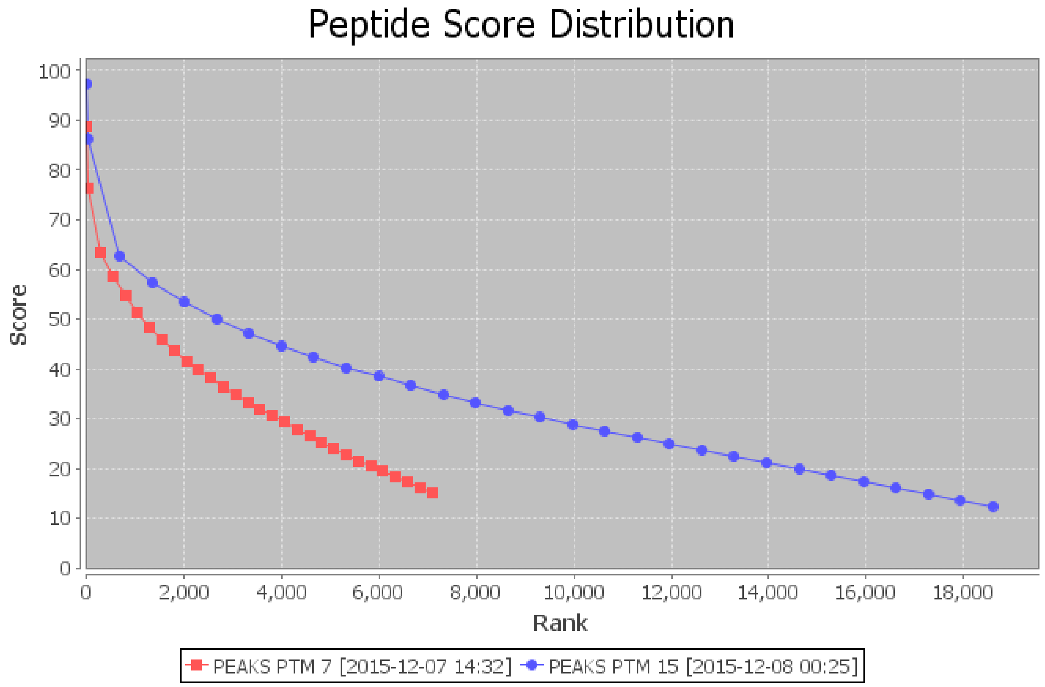

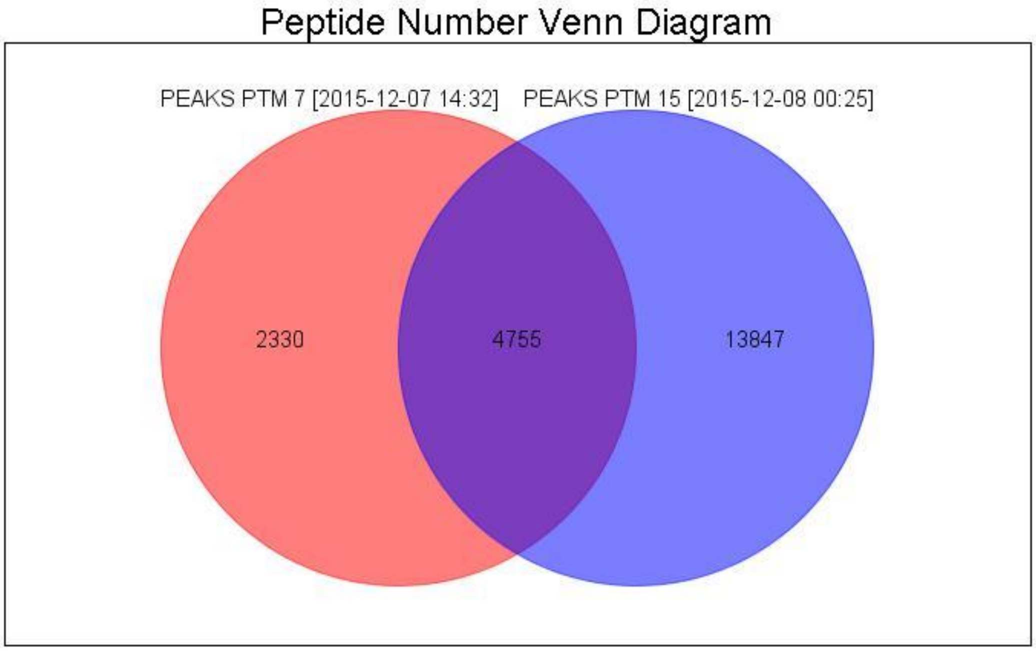

Fig A. Venn diagram of peptide score distribution and numbers of tumor and adjacent normal tissues for stage 2. (Blue colour is tumor and red colour is normal)

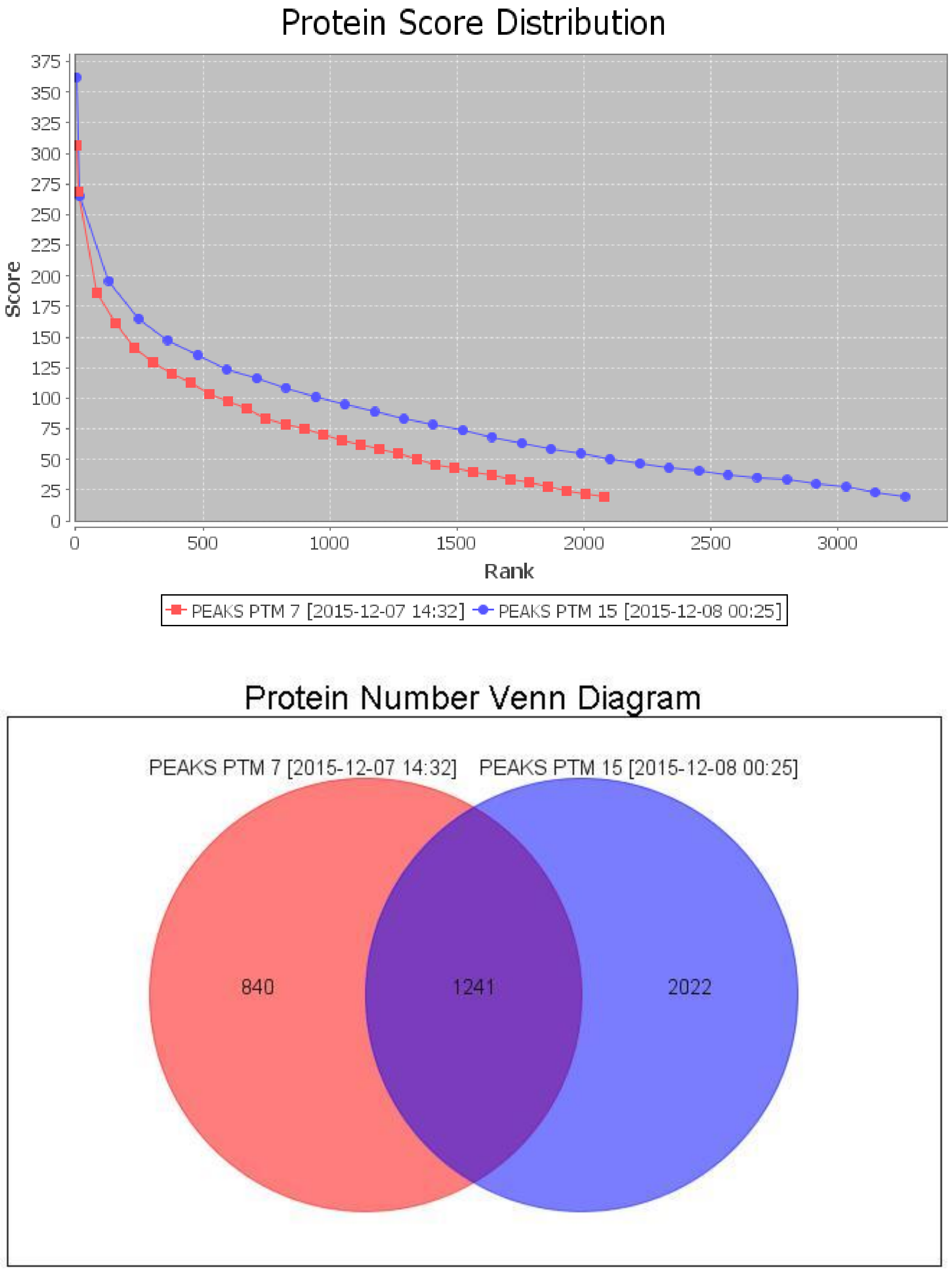

Fig B. Venn diagram of protein score distribution and protein numbers in tumor and adjacent normal tissues for stage 2.

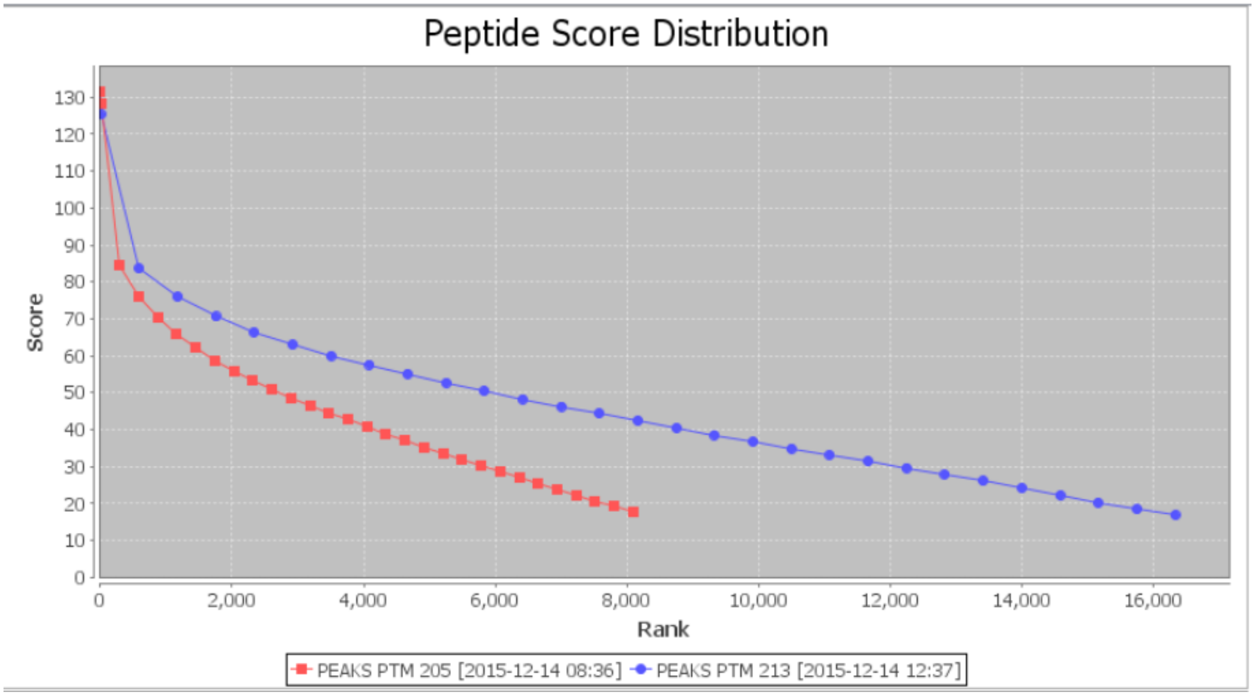

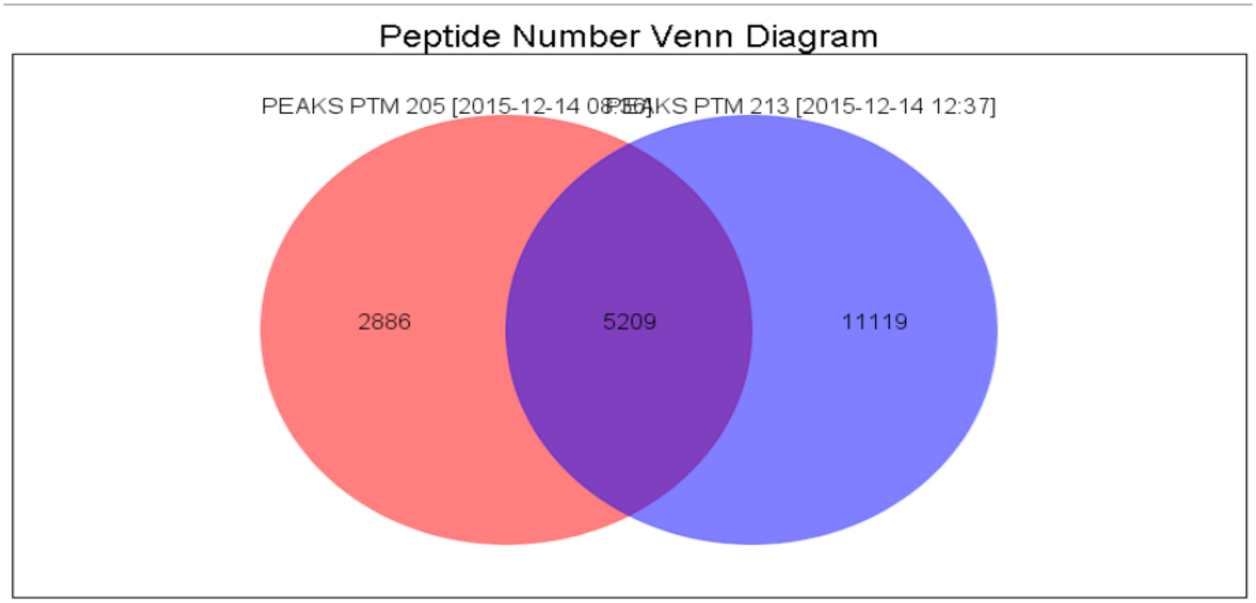

Fig C. Venn diagram of peptide score distribution and protein numbers in tumor and adjacent normal tissues for stage 3

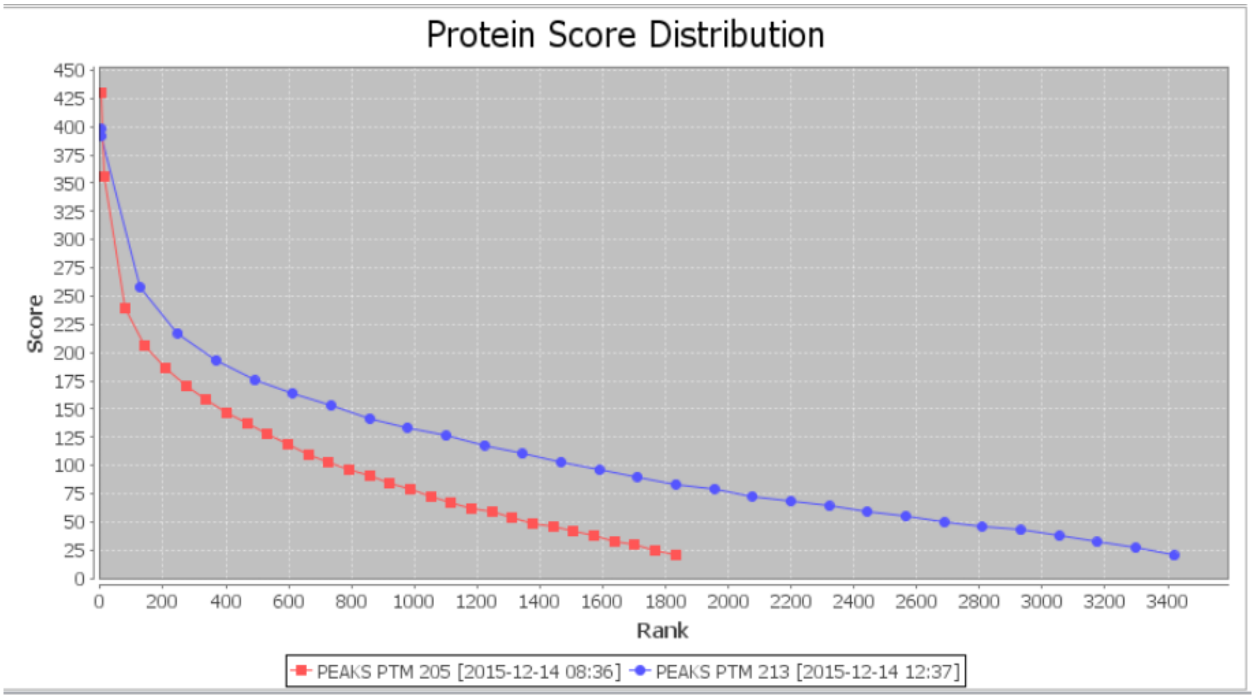

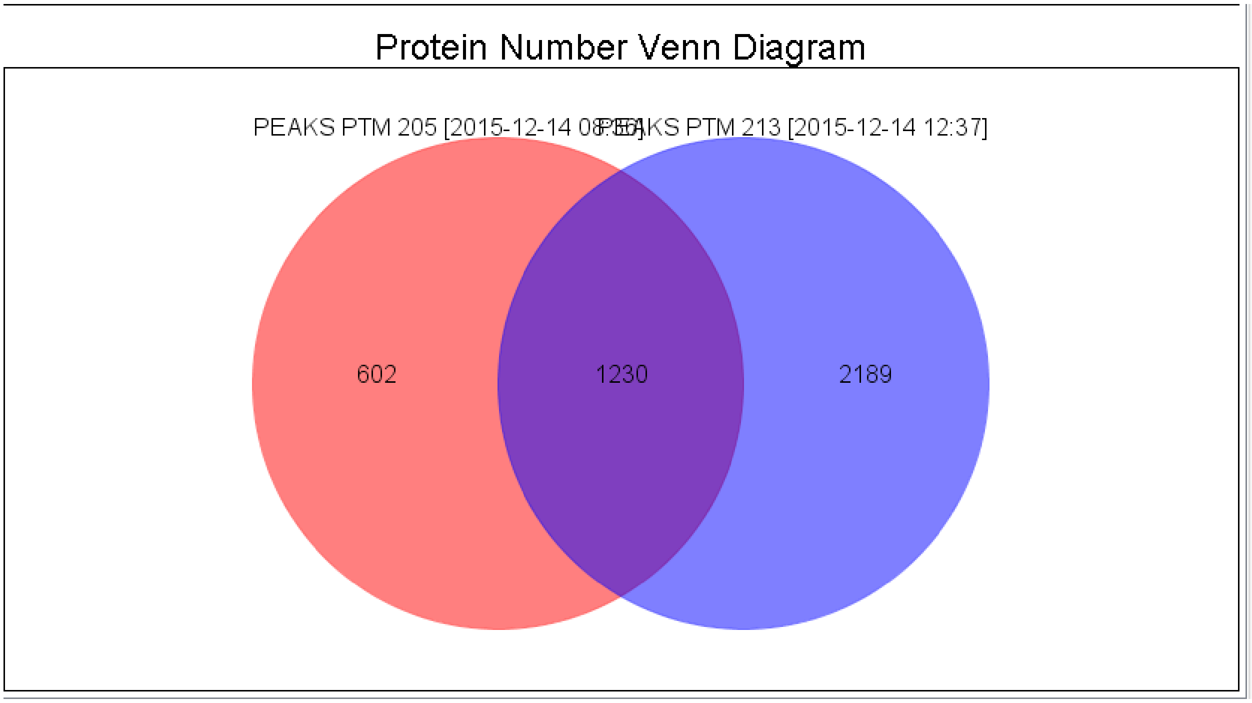

Fig D. Venn diagram of protein score distribution and protein numbers in tumor and adjacent normal tissues for stage 3
